# Tissue-specific patterns of allelically-skewed DNA methylation

**DOI:** 10.1080/15592294.2015.1127479

**Published:** 2016-01-19

**Authors:** Sarah J. Marzi, Emma L. Meaburn, Emma L. Dempster, Katie Lunnon, Jose L. Paya-Cano, Rebecca G. Smith, Manuela Volta, Claire Troakes, Leonard C. Schalkwyk, Jonathan Mill

**Affiliations:** a Institute of Psychiatry, Psychology and Neuroscience, King's College London, London, UK; b Department of Psychological Sciences, Birkbeck, University of London, London, UK; c University of Exeter Medical School, University of Exeter, Exeter, UK; d School of Biological Sciences, University of Essex, Colchester, UK

**Keywords:** Allele-specific DNA methylation, blood, brain, cerebellum, cortex, epigenetics, genomic imprinting, SNP

## Abstract

While DNA methylation is usually thought to be symmetrical across both alleles, there are some notable exceptions. Genomic imprinting and X chromosome inactivation are two well-studied sources of allele-specific methylation (ASM), but recent research has indicated a more complex pattern in which genotypic variation can be associated with allelically-skewed DNA methylation in *cis*. Given the known heterogeneity of DNA methylation across tissues and cell types we explored inter- and intra-individual variation in ASM across several regions of the human brain and whole blood from multiple individuals. Consistent with previous studies, we find widespread ASM with > 4% of the ∼220,000 loci interrogated showing evidence of allelically-skewed DNA methylation. We identify ASM flanking known imprinted regions, and show that ASM sites are enriched in DNase I hypersensitivity sites and often located in an extended genomic context of intermediate DNA methylation. We also detect examples of genotype-driven ASM, some of which are tissue-specific. These findings contribute to our understanding of the nature of differential DNA methylation across tissues and have important implications for genetic studies of complex disease. As a resource to the community, ASM patterns across each of the tissues studied are available in a searchable online database: http://epigenetics.essex.ac.uk/ASMBrainBlood.

## Introduction

DNA methylation is the most widely studied and stable epigenetic mark across the mammalian genome, playing a key role in the developmental regulation of gene expression. DNA methylation is generally symmetrical across both alleles, although exceptions characterized by allelic asymmetry include differentially methylated regions (DMRs) regulating the monoallelic expression of genes associated with X chromosome inactivation in females and genomic imprinting.^1-5^ Recently, it has been shown that the allelic-skewing of DNA methylation can also be driven by DNA sequence variation, with methylation quantitative trait loci (meQTLs) predominantly acting in *cis*.^6-11^ ASM can be regarded as a special case of intermediate DNA methylation (IM), which has been found to occur in regions spanning a large portion of the human genome. It has been estimated that ASM contributes up to 18% of IM in the human genome.^12^

DNA methylation patterns are highly dynamic during normal development and cellular differentiation^13-16^ and tissue-specific patterns of DNA methylation have been widely studied in humans.^17-20^ In complex tissues such as the brain, for example, DNA methylation differentiates between functionally distinct regions^21,22^ and cell types.^16,23-26^ Patterns of IM can also be tissue-specific,^12^ with growing evidence for the widespread prevalence of tissue-specific ASM.^27,28^ In mouse, for example, it has been reported that 28% of imprinted genes are monoallelically expressed in a single tissue type, often the brain or extra-embryonic tissue.^29^ Examples of tissue-specifically imprinted genes include *KCNQ1*, which becomes biallelically expressed in embryonic heart development,^30^ *GNAS*, which is maternally expressed in a wide-range of tissues including the anterior pituitary, thyroid and ovaries but biallelically expressed in others, such as bone and visceral adipose tissue,^31,32^ and *GRB10*, which is maternally expressed in most peripheral tissues but paternally expressed in the brain.^29,33^ Genetic influences on DNA methylation can also be tissue-specific, with meQTLs determining allelic patterns of methylation in *cis* in certain tissues or cell types.^11,34^

Increasing evidence supports a role for inter-individual variation in DNA methylation in the etiology and pathogenesis associated with a diverse range of complex disease phenotypes.^35^ Allelic differences in DNA methylation may be particularly important in this regard, acting as endophenotypes of genetic variation or additional epi-allelic layers mediating the functional consequences of genotypic variation.^36,37^ Teasing apart genetic and non-genetic effects in a tissue- and cell type-specific manner will be a crucial step in understanding the association between non-coding genetic variation, DNA methylation, and complex disease.

To investigate the role of tissue-specific variation of ASM in the human brain and its relation to allelic biases in whole blood, we examined ASM across multiple brain regions and matched blood samples collected from multiple donors. Our data shows that although a large proportion of ASM is conserved across tissues, there are specific differences in the extent and distribution of ASM sites between regions of the brain and whole blood. Genome browser tracks displaying ASM signals as well as an online tool plotting ASM for sites of interest are available for download from a searchable database (http://epigenetics.essex.ac.uk/ASMBrainBlood).

## Results

### DNA methylation is allelically-skewed at specific locations across the genome

The majority of the genome is not characterized by notable allelic biases in DNA methylation in any of the tissues assessed in this study. The array-wide average of ASM score (in absolute values) is consistently low (mean = 0.025, range = 0.023 to 0.030) (Fig. S1A, Fig. S1B and Table S1). As expected, there is, however, evidence for allelically-biased DNA methylation at a notable number of specific genomic regions; in total 9,311 (4.22%) of the 220,449 informative SNPs in our assay show evidence for allelic-skewing of DNA methylation, defined by an absolute ASM score ≥ 0.10 in at least one tissue and individual. The percentage of amplicons characterized by an ASM score ≥ 0.10 in each of the 21 profiled samples is given in Table S2. The top-ranked loci showing evidence for allelically-skewed DNA methylation in whole blood, cerebellum, and cortex (BA9), and cerebellum are listed in **Tables 1–3**. Genome Browser tracks and an online ASM database are available from our laboratory website (http://epigenetics.essex.ac.uk/ASMBrainBlood).

**Table 1.** Top 15 ASM sites in whole blood, ASM score averaged across individuals.

Rank	SNP ID	Location	Associated gene(s)	Schalkwyk et al. (2010)	Blood ASM score	Cerebellum ASM score	BA9 ASM score
1	SNP_A-2180729 (rs10276966)	7p15.2	*HIBADH, EVX1*^b^	0.29	0.25	0.01	0.03
2	SNP_A-8438077 (rs585451)	15q21.2	*ATP8B4, DTWD1*	NA	0.25	0.01	0.03
3	SNP_A-1841543 (rs10234308)	7p15.3	*MGC87042*	0.34	0.23	0.25	0.23
4	SNP_A-1946136 (rs927000)	20q13.12	*STK4*	NA	0.23	0.04	0.01
5	SNP_A-8450837 (rs4404067)	16q12.2	*SLC6A2, LPCAT2*	0.28	0.22	0.05	0.04
6	SNP_A-4279002 (rs335554)	1q41	*CENPF, KCNK2*	NA	0.22	0.08	0.09
7	SNP_A-8450539 (rs10916799)	1p36.12	*CAMK2N1, LOC339505*	0.18	0.22	0.11	0.15
8	SNP_A-8633222 (rs13165930)	5q33.3	*CCNJL*	NA	0.22	0.02	0.12
9	SNP_A-8472169 (rs11193683)	10q23.1	*NRG3*	0.01	0.22	0.09	0.07
10	SNP_A-2071005 (rs4687210)	3q28	*UTS2D*	NA	0.22	0.16	0.17
11	SNP_A-2185394 (rs852454)	7p22.1	*RNF216*	0.06	0.22	0.11	0.11
12	SNP_A-8702215 (rs12978286)	19p13.11	*FCHO1, MAP1S*	0.19	0.22	0.00	0.07
13	SNP_A-1807006 (rs10481354)	8p23.3	*CLN8, DLGAP2*^a^	0.22	0.22	0.00	0.05
14	SNP_A-8326632 (rs1542180)	2q31.1	*HOXD3, HOXD4*	NA	0.21	0.24	0.19
15	SNP_A-2008150 (rs869108)	11p15.1	*OTOG, MYOD1, USH1C*	0.22	0.21	0.16	0.07

a Known imprinted gene

b Suspected imprinted gene

**Table 2.** Top 15 ASM sites in cerebellum, ASM score averaged across individuals.

Rank	SNP ID	Location	Associated gene(s)	Cerebellum ASM score	Blood ASM score	BA9 ASM score
1	SNP_A-4255628 (rs959246)	18q12.3	*SLC14A2, SETBP1*	0.30	0.03	0.05
2	SNP_A-8696273 (rs1003533)	5q31.1	*C5orf56*	0.29	0.16	0.07
3	SNP_A-1820553 (rs7959070)	12q22	*CLLU1OS, BTG1*	0.26	0.03	0.05
4	SNP_A-1841543 (rs10234308)	7p15.3	*MGC87042*	0.25	0.23	0.23
5	SNP_A-2002282 (rs12246813)	10q22.1	*COL13A1, C10orf35*	0.24	0.10	0.10
6	SNP_A-8625237 (rs12493005)	3q26.32	*TBL1XR1*	0.24	0.20	0.24
7	SNP_A-8326632 (rs1542180)	2q31.1	*HOXD3, HOXD4*	0.24	0.21	0.19
8	SNP_A-2160121 (rs7205794)	16q23.3	*CDH13, MPHOSPH6*	0.24	0.01	0.01
9	SNP_A-2052542 (rs3098382)	5q13.2	*MAP1B*	0.24	0.04	0.19
10	SNP_A-2040586 (rs17097827)	14q32.2	*BCL11B, C14orf177*	0.23	0.10	0.18
11	SNP_A-8420373 (rs10186346)	2p22.1	*TMEM178*	0.23	0.01	0.07
12	SNP_A-1788157 (rs7158663)	14q32.2	*MEG3*^a^	0.23	0.01	0.03
13	SNP_A-4235630 (rs9641549)	7q31.2	*TFEC, MDFIC*	0.23	0.05	0.13
14	SNP_A-4264458 (rs1358229)	4q27	*TRPC3, KIAA1109*	0.23	0.01	0.00
15	SNP_A-1931666 (rs6707698)	2q34	*IKZF2, ERBB4*	0.22	0.03	0.02

a Known imprinted gene

**Table 3.** Top 15 ASM sites in cortex (BA9), ASM score averaged across individuals.

Rank	SNP ID	Location	Associated gene(s)	BA9 ASM score	Cerebellum ASM score	Blood ASM score
1	SNP_A-8625237 (rs12493005)	3q26.32	*TBL1XR1*	0.24	0.24	0.20
2	SNP_A-8463467 (rs17164474)	7q35	*OR2F2, OR2F1*	0.23	0.17	0.17
3	SNP_A-1841543 (rs10234308)	7p15.3	*MGC87042*	0.23	0.25	0.23
4	SNP_A-8652129 (rs2479084)	1p36.21	*FHAD1*	0.22	0.13	0.18
5	SNP_A-8301602 (rs398225)	3p25.3	*SRGAP3, RAD18*	0.21	0.18	0.21
6	SNP_A-2107106 (rs987377)	6q21	*AIM1*^a^, *ATG5*	0.20	0.16	0.15
7	SNP_A-2052542 (rs3098382)	5q13.2	*MAP1B*	0.19	0.24	0.04
8	SNP_A-2307481 (rs716591)	15q26.2	*LOC400456, MCTP2*	0.19	0.21	0.17
9	SNP_A-8643280 (rs3121125)	1q21.1	*HFE2, NBPF10*	0.19	0.17	0.15
10	SNP_A-8326632 (rs1542180)	2q31.1	*HOXD3, HOXD4*	0.19	0.24	0.21
11	SNP_A-1998023 (rs9722212)	9q34.11	*TOR1B, PTGES*	0.19	0.15	0.19
12	SNP_A-8640607 (rs12632177)	3q27.1	*MCF2L2*	0.18	0.11	0.07
13	SNP_A-4291638 (rs12670584)	7p13	*YKT6*	0.18	0.19	0.01
14	SNP_A-1892234 (rs17303015)	5p12	*MGC42105*	0.18	0.11	0.03
15	SNP_A-8653671 (rs4871852)	8p21.3	*TNFRSF10D*	0.18	0.09	0.11

a Known imprinted gene

### Patterns of ASM in whole blood overlap with those identified in a previous study

In a previous study we characterized allelically-skewed DNA methylation in whole blood derived from 5 monozygotic twin pairs.^7^ There is a highly significant correlation between absolute ASM scores across all probes informative in both data sets (n = 129,559, *r* = 0.21, *P* < 1.0 × 10^−50^, Fig. S2), even though the majority of the probes do not exhibit ASM. Of the 2,704 ASM loci identified in Schalkwyk et al., 1,717 (63.50%) are informative in the current study, with a highly significant cross-study correlation of ASM scores at these probes (*r* = 0.52, *P* < 1.0 × 10^−50^). Likewise, there is a highly significant correlation between ASM scores across the two studies at sites showing allelically-skewed DNA methylation in blood in the current study which were also informative in our previous study (r = 0.38, P = 3.0 × 10-28). Of the 15 top-ranked blood ASM sites identified in our current study (**Table 1**), 7 of the 9 sites (78%) also informative in our previous study of ASM in blood^7^ were characterized by an absolute ASM score ≥ 0.10 in both analyses. These data confirm the validity of the MSNP approach for identifying allelically-skewed DNA methylation, reinforcing our previous conclusions about the extent of ASM in whole blood.^7^

### The extent and distribution of ASM differs across tissues

**Figure 1.**
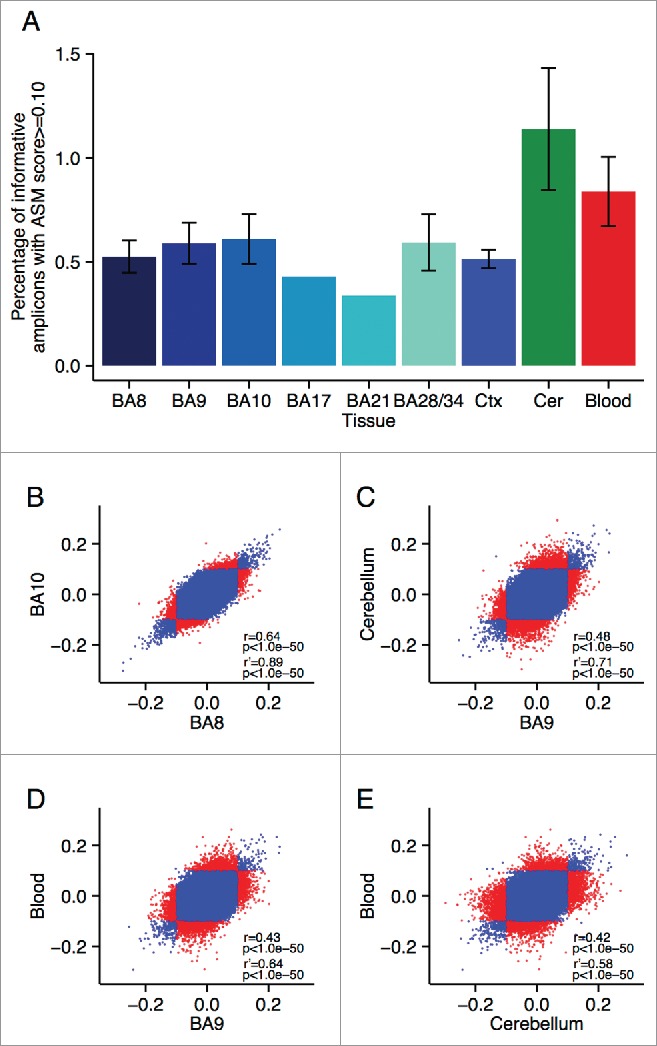
Allelic-skewing is less prevalent and less variable across cortical regions compared to cerebellum and whole blood. (A) The average proportion of informative amplicons showing an ASM score ≥ 0.10 for the 6 cortical regions profiled as well as averaged across the cortical areas (Ctx) is consistently lower (ASM score range = 0.34–0.61%) than in cerebellum (Cer) (ASM prevalence = 1.14%) or blood (ASM prevalence = 0.84%). Standard errors are shown for tissues for which samples were available from all 3 individuals. (B) – (E) Correlations of ASM scores are shown with each point representing one probe in one individual. Probes classified as allelically-skewed at an ASM score ≥ 0.10 in only one of the 2 compared tissues are highlighted in red. A higher degree of between-tissue variability is observed between cerebellum, cortex, and whole blood than between different cortical regions (shown as an example is BA8 vs. BA10). This difference becomes even more pronounced when restricting the set of probes to those that show allelic-skewing at an ASM score ≥ 0.10 in at least one of the 2 compared tissues (see subset correlation *r*’).

**Figure 2.**
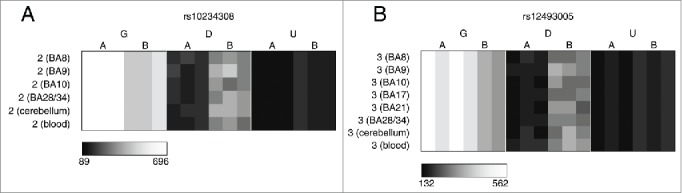
Multiple loci are characterized by consistent allelic-skewing of DNA methylation across all tissues. Heatmaps display allele signal intensities for genomic DNA (G), MSRE-digested DNA (D) and fully unmethylated, MSRE-digested DNA (U) in all tissues. A and B denote the 2 alleles of the SNP and brightness represents the quantile normalized signal intensity, with the scale displayed below the heatmap. Shown are the 2 top-ranked probes characterized by consistent ASM across tissues. These two probes were informative in (A) individual 2 for rs10234308 and (B) individual 3 for rs12493005.

**Figure 3.**
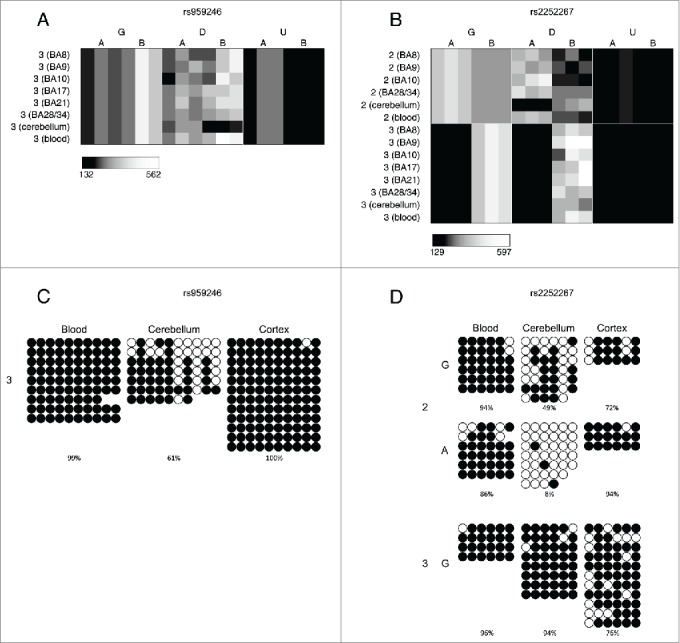
A number of loci are characterized by allelic-skewing of DNA methylation in only one tissue with minimal ASM present in any of the other tissues examined. Heatmaps display allele signal intensities for genomic DNA (G), MSRE-digested DNA (D) and fully unmethylated, MSRE-digested DNA (U) in all tissues. A and B denote the 2 alleles of the SNP and brightness represents the quantile normalized signal intensity, with the scale displayed below the heatmap. Two of the top-ranked cerebellum-specific ASM signals are (A) rs959246 (informative for individual 3) and (B) rs2252267 (informative for individual 2). The tissue-specific patterns of DNA methylation in these 2 loci were validated by clonal bisulfite sequencing, confirming the findings from the MSNP assay (C,D). Each row represents a single DNA molecule, with black dots depicting methylated cytosines and white dots depicting unmethylated cytosines. The percentage of methylated cytosines for each sample is displayed below the plots. The amplicon spanning the DMR associated with rs959246 in (C) did not encompass a SNP enabling us to distinguish between the 2 alleles, however the methylation pattern shows evidence for tissue-specific intermediate methylation (IM) in the cerebellum. G and A in (D) denote the 2 alleles determined by SNP variation within the amplicon in heterozygous individual 2. Cerebellum-specific hypomethylation of the A allele is observed surrounding rs2252267, with individual 3 (homozygous for the G allele) being highly methylated in all 3 tissues.

The average proportion of informative sites characterized by allelically-skewed DNA methylation (absolute ASM score ≥ 0.10) in each of the 8 tissues profiled was examined (**Fig. 1A**). **Table 4** lists the top-ranked consistently allelically-skewed probes across cerebellum, whole blood, and cortex (BA9), with specific examples shown in **Fig. 2A and Fig. 2B**. Allelically-skewed DNA methylation appears to be consistently less prevalent in cortical regions (informative probes with ASM score ≥ 0.10 = 0.54%) compared to the cerebellum (1.14%) and whole blood (0.84%). The elevated level of allelically-skewed DNA methylation in the cerebellum and whole blood relative to cortex is more pronounced at more extreme ASM score thresholds (i.e., ASM score ≥ 0.20, cortex = 0.003%, cerebellum = 0.019%, whole blood = 0.013%) (Fig. S1C, Fig. S1D and Fig. S3). Of note, there is little variation in the prevalence and distribution of ASM scores between different regions of the cortex (average correlation between 2 cortical areas = 0.52, **Fig. 1B** and Fig. S4). We therefore selected one representative cortical region (BA9) for inclusion in subsequent analyses. In contrast, we find more striking differences between cortex, cerebellum and whole blood samples with inter-tissue correlations ranging from *r* = 0.42 to 0.48 (**Figs. 1C-E**). **Table 5** lists the probes showing the highest level of variation in ASM scores across tissues with specific examples shown in **Fig. 3A and Fig. 3B**. We used clonal bisulfite sequencing to validate tissue-specific ASM identified by the MSNP method in these 2 regions (**Fig. 3C and Fig. 3D**), confirming the patterns observed in our array data for both loci.

**Table 4.** Top 15 loci characterized by consistent ASM across cerebellum, whole blood, and cortex (BA9).

Rank	SNP ID	Location	Associated gene(s)	Cerebellum ASM score	Blood ASM score	BA9 ASM score	Tissue average
1	SNP_A-1841543 (rs10234308)	7p15.3	*MGC87042*	0.25	0.23	0.23	0.24
2	SNP_A-8625237 (rs12493005)	3q26.32	*TBL1XR1*	0.24	0.20	0.24	0.22
3	SNP_A-8326632 (rs1542180)	2q31.1	*HOXD3, HOXD4*	0.24	0.21	0.19	0.21
4	SNP_A-8301602 (rs398225)	3p25.3	*SRGAP3, RAD18*	0.18	0.21	0.21	0.20
5	SNP_A-8463467 (rs17164474)	7q35	*OR2F2, OR2F1*	0.17	0.17	0.23	0.19
6	SNP_A-2307481 (rs716591)	15q26.2	*MCTP2, LOC400456*	0.21	0.17	0.19	0.19
7	SNP_A-1894705 (rs986324)	Xp22.11	*DDX53, ZNF645*	0.18	0.20	0.17	0.18
8	SNP_A-2071005 (rs4687210)	3q28	*UTS2D*	0.16	0.22	0.17	0.18
9	SNP_A-1998023 (rs9722212)	9q34.11	*PTGES, TOR1B*	0.15	0.19	0.19	0.18
10	SNP_A-8652129 (rs2479084)	1p36.21	*FHAD1*	0.13	0.18	0.22	0.18
11	SNP_A-8696273 (rs1003533)	5q31.1	*C5orf56*	0.29	0.16	0.07	0.17
12	SNP_A-1862496 (rs17578280)	1p31.1	*LRRIQ3, NEGR1*	0.18	0.17	0.17	0.17
13	SNP_A-2040586 (rs17097827)	14q32.2	*C14orf177, BCL11B*	0.23	0.10	0.18	0.17
14	SNP_A-1932077 (rs220030)	15q11.2	*SNRPN*^a^	0.18	0.18	0.15	0.17
15	SNP_A-2107106 (rs987377)	6q21	*AIM1*^a^, *ATG5*	0.16	0.15	0.20	0.17

a Known imprinted gene

**Table 5.** Top 15 tissue-specific ASM sites, defined by highly variable ASM scores across cerebellum, whole blood, and cortex (BA9).

Rank	SNP ID	Location	Associated gene (s)	Cerebellum ASM score	Blood ASM score	BA9 ASM score	SD
1	SNP_A-4255628 (rs959246)	18q12.3	*SLC14A2, SETBP1*	−0.30	−0.03	−0.05	0.15
2	SNP_A-1946136 (rs927000)	20q13.12	*STK4*	−0.04	0.23	0.01	0.15
3	SNP_A-8438077 (rs585451)	15q21.2	*ATP8B4, DTWD1*	0.01	−0.25	0.00	0.15
4	SNP_A-8397727 (rs1123514)	5q13.3	*ENC1, RGNEF*	−0.16	0.11	−0.01	0.13
5	SNP_A-2273834 (rs2252267)	14q23.1	*PRKCH*	0.19	−0.03	−0.05	0.13
6	SNP_A-4264458 (rs1358229)	4q27	*TRPC3, KIAA1109*	−0.23	0.01	0.00	0.13
7	SNP_A-8643616 (rs11700515)	21q22.3	*COL6A1, PCBP3*	0.20	−0.01	−0.03	0.13
8	SNP_A-2180729 (rs10276966)	7p15.2	*HIBADH, EVX1*^b^	−0.01	−0.25	−0.03	0.13
9	SNP_A-4210659 (rs10517764)	4q32.2	*NAF1, FSTL5*	0.07	−0.14	−0.15	0.13
10	SNP_A-1997061 (rs10512149)	9q21.33	*SLC28A3, NTRK2*	−0.19	0.02	0.05	0.13
11	SNP_A-2160121 (rs7205794)	16q23.3	*CDH13, MPHOSPH6*	−0.24	−0.01	−0.01	0.13
12	SNP_A-8667432 (rs519782)	1p36.11	*C1orf201*	0.19	−0.04	−0.01	0.13
13	SNP_A-1787058 (rs10951911)	7p12.3	*TNS3, C7orf65*	−0.20	−0.01	0.04	0.13
14	SNP_A-1931666 (rs6707698)	2q34	*IKZF2, ERBB4*	−0.22	−0.03	0.02	0.13
15	SNP_A-8502638 (rs10989120)	9q31.1	*TMEFF1, C9orf30*	0.04	−0.20	−0.15	0.13

b Suspected imprinted gene

### Informative MSNP probes within DNase I hypersensitive regions are characterized by elevated ASM scores

Enrichment analyses were performed using a Kruskal-Wallis rank-sum test for ASM rank differences between the annotated genic regions (see **Materials and Methods**). We observed a differential distribution of ASM scores across annotated genic regions (i.e., coding, 5′UTR, intergenic, intron, promoter, 3′UTR) in cortex (BA9) (*P* = 1.29 ×10^−15^), cerebellum (*P* = 3.98 ×10^−14^), whole blood (*P* = 2.06 ×10^−12^), and the cross-tissue analysis (*P* = 2.12 ×10^−26^). Post-hoc tests identified these differences to be primarily driven by an enrichment of high ASM scores in promoter regions (Fig. S5). We used data from ENCODE^38^ to assess whether ASM is enriched in regions associated with DNase I hypersensitive (DHS) sites identified in multiple tissues including frontal cortex and cerebellum, as well as CD14+ monocytes and naïve B cells (as a proxy for blood). We compared the ASM score ranks for informative probes between regions defined by the presence or absence of DHS sites using a Wilcoxon rank-sum test (see **Materials and Methods**). DHS peaks across all tissues are enriched for higher ASM scores identified in cortex (BA9), cerebellum, and whole blood (Table S3 and Fig. S6). Of note, the most striking enrichment is found for cerebellum ASM scores in regions characterized by cerebellum DHS peaks in ENCODE (*P* = 3.51 × 10^−220^).

### Inter-individual variation in ASM

**Figure 4.**
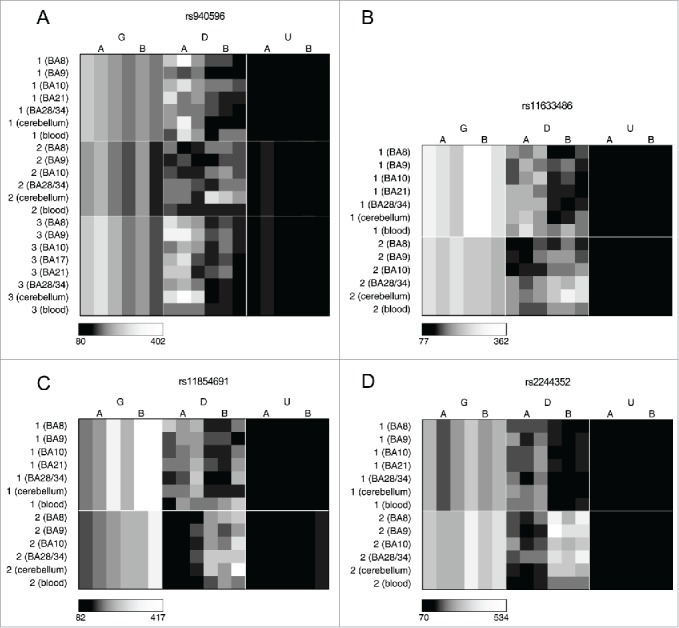
Cases of allelic-flipping of DNA methylation between individuals are found both in known imprinted gene clusters as well as regions not previously confirmed as being imprinted. The heatmaps show allele signal intensities for genomic DNA (G), MSRE-digested DNA (D) and fully unmethylated, MSRE-digested DNA (U) in all tissues. A and B denote the 2 alleles of the SNP and brightness represents the quantile normalized signal intensity, with the scale displayed below the heatmap. (A)-(C) Three probes in the vicinity of the known imprinted cluster on chromosome 15q11.2 show variable ASM and allelic-flipping in cerebellum [(A) rs940596, (B) rs11633486, (C) rs11854691]. (D) Allelic-flipping of DNA methylation across all tissues is observed in the vicinity of WRB (rs2244352), which has not been reported as imprinted previously.

We next examined inter-individual differences in ASM score at specific loci, defining probes with a large range of ASM scores across the 3 individuals as being characterized by “variable ASM.” Differentially methylated regions (DMRs) associated with genomic imprinting, for example, are characterized by parental-origin-specific ASM and are expected to show consistently large ASM scores that exhibit allelic-flipping, resulting from genotype-independent ASM. Genotype-driven ASM, in contrast, is likely to be exemplified by consistent allelic biases in DNA methylation across individuals, and is generally not variable between individuals of the same genotype. Fig. S7 shows the correlation in ASM scores across the 3 individuals profiled by MSNP, with tissue-specific correlations given in Table S4. As expected, the individuals were more highly correlated for loci characterized by high ASM scores. For probes informative in at least 2 individuals we examined the range of ASM scores across individuals and identified the top ranked variable ASM probes in each tissue (Table S5-S7) as well as cross-tissue variable sites, which show consistent inter-individual variation across all tissues (**Table 6**). Some sites show evidence of allelic-flipping in ASM score between individuals, indicative of genomic imprinting. These included several probes in the vicinity of the imprinted gene cluster on chromosome 15q11.2 (**Figs. 4A-C**). High ASM scores were also observed in the vicinity of other known imprinted loci, for example, *SNRPN* (Fig. S8A), *DLGAP2* (Fig. S8B), *AIM1* (Fig. S8C), *MEG3* (Fig. S8D), *BLCAP* (Fig. S8E), and *GRB10* (Fig. S8F), in addition to loci suspected to be imprinted, e.g., *TRAPPC9* (Fig. S8G), *EVX1* (Fig. S8H), and *TGFBI*/*VTRNA2* (Fig. S8I); however, we were unable to examine variable ASM in many of these regions because they were only informative (i.e., heterozygous) in a single individual. Notably, we also identified allelic-flipping in the vicinity of loci not previously characterized as being imprinted, for example *WRB* (**Fig. 4D**) and *ITPKI* (Fig. S9). Other variable ASM sites are marked by both high and low ASM scores in different individuals, rather than allelic-flipping between them, for example, *MGST3*/*LOC400794* (Table S5). Interestingly, we identified a number of sites characterized by tissue-specific variable ASM. A notable example is the imprinted gene *GRB10*, which has been previously shown to be differentially maternally- and paternally-expressed in a tissue-specific manner^29,33^ (Fig. S8F).

**Table 6.** Top 15 variable ASM sites, defined by average range of ASM scores between individuals across cerebellum, whole blood, and cortex (BA9).

Rank	SNP ID	Location	Associated gene(s)	Cerebellum range	Blood range	BA9 range	Average range
1	SNP_A-8579417 (rs1538116)	1q24.1	*MGST3, LOC400794*	0.24	0.35	0.32	0.30
2	SNP_A-2019421 (rs2244352)	21q22.2	*WRB*	0.36	0.24	0.29	0.30
3	SNP_A-4219174 (rs2346019)	5q31.1	*TGFBI, VTRNA2*^b^	0.23	0.23	0.30	0.25
4	SNP_A-4208914 (rs927651)	20q13.2	*CYP24A1*	0.25	0.23	0.17	0.22
5	SNP_A-8717059 (rs12713666)	2p13.3	*ARHGAP25*	0.26	0.15	0.21	0.21
6	SNP_A-8692937 (rs4605656)	4q24	*CXXC4, TACR3*	0.16	0.18	0.25	0.20
7	SNP_A-8634251 (rs1209228)	14q24.2	*RGS6, SIPA1L1*	0.19	0.15	0.24	0.19
8	SNP_A-8329713 (rs16825906)	3q13.31	*LSAMP, IGSF11, LOC285194*	0.21	0.13	0.22	0.19
9	SNP_A-8713358 (rs16890883)	4p15.33	*CPEB2, LOC152742*	0.24	0.14	0.17	0.19
10	SNP_A-8638348 (rs4525744)	2p21	*SRBD1*	0.20	0.18	0.17	0.18
11	SNP_A-1855770 (rs3764124)	13q34	*CUL4A*	0.16	0.23	0.15	0.18
12	SNP_A-4259064 (rs7766133)	6p22.3	*MBOAT1*	0.17	0.17	0.20	0.18
13	SNP_A-2118217 (rs1695824)	1p36.33	*VWA1, TMEM88B*	0.25	0.16	0.13	0.18
14	SNP_A-1880775 (rs6116750)	20p12.3	*PROKR2*	0.23	0.13	0.18	0.18
15	SNP_A-8424056 (rs3922835)	18q12.1	*CDH2*^b^, *CHST9*	0.18	0.04	0.32	0.18

b Suspected imprinted gene

### Variable ASM sites are flanked by extended regions of intermediate DNA methylation

**Figure 5.**
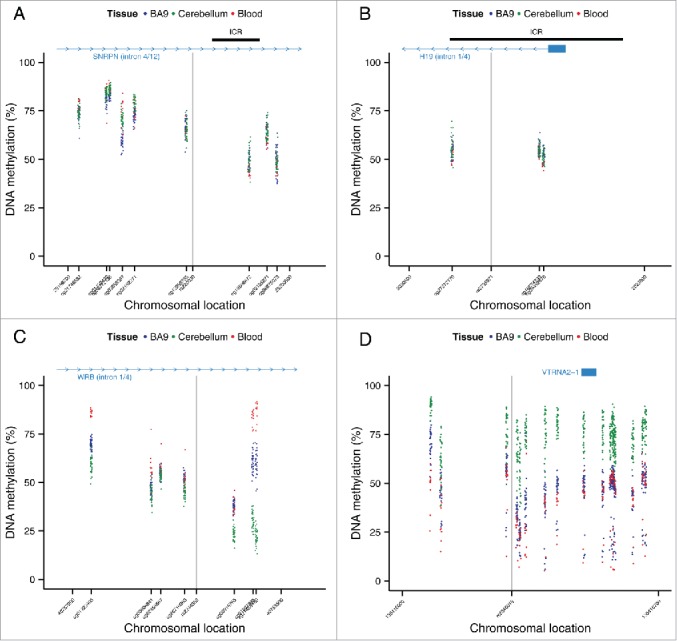
Regions around variable ASM sites are enriched for genomic domains characterized by intermediate DNA methylation. Flanking regions (1 kb) of variable ASM sites show a significant enrichment in intermediately methylated probes on the Illumina 450K Human Methylation Array (*P* range = 6.82 × 10^−11^ – 0.005). The scatter plots (A)-(D) show the location of genes and imprinting control regions (ICR) if overlapping the plotting window, as well as the location of the SNP from the MSNP assay (gray vertical line). These intermediate DNA methylation patterns span several known imprinted regions, for example (A) a flanking region of rs220030 in the *SNRPN* imprinted DMR, and (B) a region around rs2735971 in the imprinted gene *H19*. (C) Other sites showing allelic-flipping and intermediately methylated flanking regions lie in areas not previously known to be imprinted, for example rs2244352, which lies in an intron of *WRB*. (D) A polymorphic ASM pattern is observed in a flanking region around rs2346019, a downstream gene variant *of VTRNA2-1*, in which the majority of samples display intermediate DNA methylation in cortex (BA9) and whole blood. Of note, 4 samples show consistent hypomethylation across this region.

**Figure 6.**
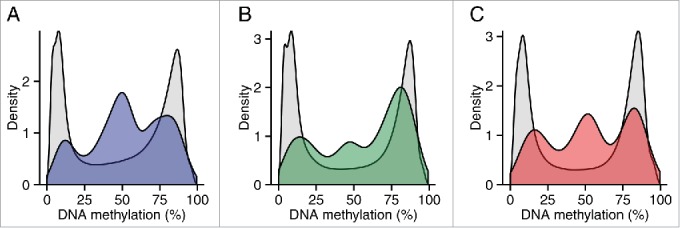
Regions around variable ASM are enriched in intermediate DNA methylation. The distributions of DNA methylation at the 65 Illumina 450K Human Methylation Array probes within 1 kb of the top 100 variable ASM sites (Table S14) show an enrichment in intermediately methylated probes compared to DNA methylation levels across the whole array (shown in gray) in (A) cortex (BA9), (B) cerebellum, and (C) whole blood).

We next quantified genome-wide patterns of DNA methylation in a larger sample (n=39) of matched whole blood, cortex (BA9), and cerebellum samples using the Illumina Infinium HumanMethylation450 BeadChip (450K array). For the 100 top-ranked ASM sites in each of the 3 tissues, plus the 100 top-ranked cross-tissue, tissue-specific, and variable ASM sites we identified probes on the array located within 1 kb of the ASM marker SNPs (Table S8; detailed in Table S9–S17) to investigate patterns of DNA methylation across an extended region. As expected, regions around known imprinted loci identified by our ASM analysis are flanked by extended regions of intermediate DNA methylation (i.e., average levels of DNA methylation between 0.4 and 0.6) (**Fig. 5A and Fig. 5B**). We observe a highly significant enrichment (*P* range = 6.82 × 10^−11^ – 0.005) of intermediate DNA methylation relative to overall levels identified on the 450K array in regions flanking variable ASM sites in all 3 tissues (**Table 7** and **Fig. 6**). For example, intermediate DNA methylation was observed around the variable ASM site overlapping *WRB* (**Fig. 5C**), that showed evidence of allelic-flipping (**Fig. 4D**). Another probe exhibiting variable ASM annotated to *TGFBI/VTRNA2-1* on chromosome 5 also shows a similar pattern of intermediate DNA methylation (**Fig. 5D**). Interestingly, 4 individuals are distinguished by consistent hypomethylation in whole blood and cortex across 16 of the 19 sites, consistent with previous reports describing polymorphic imprinting of this locus.^39,40^

**Table 7.** Enrichment in intermediate methylation (IM) near ASM regions.

	IM rate	O/E	*P*
Blood^d^	5.88%	0.98	0.576
Cerebellum^d^	11.49%	1.75	0.059
BA9^d^	13.29%	1.45	0.065
Cross-tissue	8.11%	0.87	0.741
Tissue-specific	8.00%	0.85	0.701
Variable	38.46%	4.10	9.56 × 10^−11^
Variable (BA9)^d^	37.50%	4.09	8.86 × 10^−9^
Variable (cerebellum)^d^	16.95%	2.58	0.005
Variable (blood)^d^	34.48%	5.77	6.82 × 10^−11^

c IM is defined as an average methylation between 0.4 and 0.6, enrichment *P* values are based on a hypergeometric test based on the background distribution of IM.

d We used tissue-specific background distributions for ASM types based on single tissues.

### Identification of tissue-specific genotype-driven ASM

**Figure 7.**
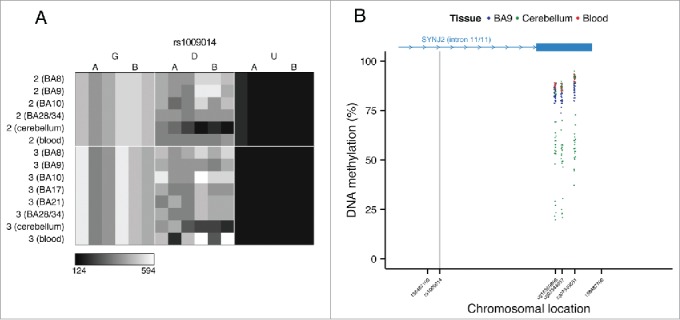
Genotype-driven ASM can be tissue-specific. (A) The tissue-specific ASM site rs1009014, located in an intron of *SYNJ2*, shows allelic-skewing of DNA methylation in cerebellum (ASM score = 0.16) but not in cortex (ASM score = 0.04) or whole blood (ASM score = 0.03). (B) DNA methylation levels for Illumina 450K Human Methylation Array probes in cerebellum across a flanking region of this locus exhibit a genotype-driven tissue-specific DNA methylation pattern. The scatter plot shows the location of *SYNJ2* (transcript variant 1), as well as the location of the informative SNP from the MSNP assay (gray vertical line).

In contrast to the intermediate DNA methylation patterns enriched in the vicinity of variable ASM sites, non-variable ASM (i.e., characterized by consistent ASM scores across individuals) is not significantly enriched for intermediate DNA methylation and in some cases is exemplified by trimodal patterns of DNA methylation, which are highly suggestive of genotype-driven ASM acting in *cis*. Of note, we also observe examples of tissue-specific genotype-driven ASM. For example, a tissue-specific ASM site identified as showing allelically-skewed DNA methylation in cerebellum (ASM score = 0.16) but not whole blood (ASM score = 0.03) or cortex (ASM score = 0.04) (**Fig. 7A**) located in an intron of the gene *SYNJ2* is flanked by trimodal levels of DNA methylation in cerebellum but not blood or cortex (**Fig. 7B**).

## Discussion

This study confirms the relatively widespread distribution of allelically-skewed DNA methylation in the human genome, corroborating our previous data generated in whole blood.^7^ We also present evidence for tissue-specific differences in the quantity and distribution of ASM between different regions of the human brain, and between brain and whole blood. Our findings are in line with previous reports, confirming the importance of tissue-specific DNA methylation profiles across the brain.^22,41^

Although our data confirm previous studies, identifying more between-tissue variation than inter-individual variation,^28^ we find clear examples where ASM is variable between individuals. While the number of samples profiled in this study is too small to accurately determine how much of the observed inter-individual variation in ASM results from genetic and non-genetic effects, previous studies suggest that the majority of such variation is likely to be genetically driven.^7,8,11^ Interestingly, we identify instances of tissue-specific allelically-skewed DNA methylation resulting from both genomic imprinting and genotypic effects. For example, we observe tissue-specific variable ASM around the imprinted growth factor receptor-bound protein 10 gene (*GRB10*), which encodes a protein that interacts with insulin-like growth factors^42,43^ and we observe genotype-driven ASM exclusively in cerebellum for several probes within the synaptojanin 2 gene (*SYNJ2*), which encodes a protein involved in the uncoating of vesicles.^44,45^ Such tissue-specific ASM has important implications for epigenetic epidemiology, and provides a mechanism by which genotype may exert an effect on gene function and regulation in a tissue-specific manner.

This study has a number of important limitations. First, although we used a unique set of samples comprising of matched tissues obtained from the same donors, the number of individuals profiled in our analysis was small, meaning we cannot definitively distinguish between genetic and non-genetic effects, or make broad statements about general patterns of inter-individual variation of ASM. Our Illumina 450K array validation studies were undertaken in a larger set of individuals, but could only confirm intermediate levels of DNA methylation and not detect allele-specific patterns. Second, given the limited availability of RNA from the same samples, we were unable to relate our ASM findings to allelic patterns of gene expression in the same individuals. Previous studies, however, have shown that ASM is linked to allele-specific expression of nearby genes.^28^ Third, our analyses were undertaken on whole tissue, and represent aggregate values across a number of individual cell types. Fourth, the MSNP approach utilizes SNP microarrays—these do not interrogate the whole genome, and can only assess pools of DNA molecules. Allelic patterns of DNA methylation across individual DNA molecules cannot be directly assessed using this approach, as would be possible using bisulfite-sequencing methods. Furthermore, our threshold for calling allelic imbalances in DNA methylation is somewhat arbitrary; it is likely that our data is confounded by both false positives and negatives. We did, however, find very consistent overlap in whole blood ASM data with that reported in our previous study using the same laboratory and analysis methods,^7^ confirming the validity of the MSNP approach. Furthermore, we validated our findings using 2 independent platforms: clonal bisulfite sequencing and the Illumina 450K Human methylation array. Using the latter, we were able to show that variable ASM sites are located in an extended context of intermediate DNA methylation, supporting a regional regulatory role of DNA methylation in these domains, which is potentially driving intermediate expression levels in a quantitative manner across gene regulation clusters.^12^ In addition, we observed a significant enrichment of ASM in regions characterized by DHS peaks across several tissues. This enrichment of ASM in the vicinity of markers of open chromatin supports the involvement of ASM in transcriptional activity.

To conclude, we explored inter- and intra-individual variation in ASM across several regions of the human brain and whole blood from multiple individuals. Consistent with previous studies, we find relatively widespread ASM, observing allelically-skewed DNA methylation flanking known imprinted regions, and show that ASM sites are often located in an extended genomic context of intermediate DNA methylation. Interestingly, we detect cases of genotype-driven ASM, which are also tissue-specific. These findings contribute to our understanding about the nature of differential DNA methylation across tissues and have important implications for genetic studies of complex disease. As a resource to the community, ASM patterns across each of the tissues studied are available in a searchable online database: http://epigenetics.essex.ac.uk/ASMBrainBlood.

## Materials and methods

### Genome-wide analysis of allelically-skewed DNA methylation

Post-mortem brain and pre-mortem whole blood samples from 2 female and one male donors were provided by the MRC London Neurodegenerative Disease Brain Bank (http://www.kcl.ac.uk/ioppn/depts/bcn/Our-research/Neurodegeneration/brain-bank.aspx). Subjects were approached in life for written consent for brain banking, and all tissue donations were collected and stored following legal and ethical guidelines (NHS reference number 08/MRE09/38; the HTA license number for the LBBND brain bank is 12293). All samples were free from neuropathological and neuropsychiatric disease. A detailed list of brain regions obtained for each individual is provided in Table S18. Genomic DNA was isolated from all tissue samples using a standard phenol-chloroform protocol and assessed for purity and degradation prior to analysis (see Davies et al.^22^ for additional information about the samples used in this study). The MSNP method, described previously,^6,7^ was used to quantitatively assess allelic-skewing of DNA methylation across the genome. Briefly, Affymetrix Genome-wide Human SNP 6.0 Arrays were used to genotype a) DNA from each tissue sample digested with a cocktail of MSREs (HpaII: 5′ -Cˆ C G G-3′, HhaI: 5′ -G C Gˆ C-3′, and AciI: 5′ -C ˆ C G C-3′) (D arrays), b) unmethylated whole-genome-amplified DNA for each individual digested with the same cocktail of MSREs to control for possible confounding effects of DNA sequence polymorphisms located at MSRE cut-sites (U arrays), and c) genomic DNA from each of the 3 individuals to identify heterozygous (informative) SNPs (G arrays). Unmethylated DNA was produced by whole-genome amplifying 100 ng cerebellum DNA using the Qiagen RepliG kit (Qiagen, Crawley, UK) using the manufacturer's protocol. In total 28 genotyping arrays were processed: 22 D arrays (DNA from between 6 and 7 brain regions plus whole blood, for each individual), 3 U arrays (one for each individual), and 3 G arrays (one for each individual). Additional methodological details are available in Schalkwyk et al.^7^

### Selection of informative SNPs and quantification of ASM

To be informative in the ASM assay, SNPs must be heterozygous, and the amplicon must contain an MSRE cut site.^6,7^ To guard against poorly performing SNP probes we also removed consistently low signal intensity SNPs across the 22 G arrays, and those yielding a highly variable U/G signal ratio (SD > 0.077) across all samples. A total of 220,449 SNPs passed our stringent filtering criteria and were classified as informative and heterozygous in at least one individual. The number of informative SNPs in each of the individual samples profiled by the MSNP method is shown in Table S19. Quantitative measures of ASM were derived by comparing signal intensities between the D (MSRE digested) and G (genomic DNA) arrays using the SNPMaP package (v1.02) in R that was developed for the estimation of allele frequencies in DNA pools genotyped on SNP arrays.^46^ Briefly, relative allele score (RAS) values were generated for all SNPs on the array, which are defined as A/(A + B), where A and B are the intensities of the probes for the 2 alleles of a given SNP. For a given SNP in a heterozygous individual, ASM (or allelic-skewing of DNA methylation) is detected as a difference in RAS between the G and D arrays. We call this difference in RAS “ASM score” and define probes showing an absolute ASM score ≥ 0.10 as “allelically-skewed.” A UCSC custom annotation track showing the location of all 220,449 loci and the degree of allelic-skewing in DNA methylation across each tissue and individual is available for download from our website (http://epigenetics.essex.ac.uk/ASMBrainBlood). Enrichment analyses were performed using a Kruskal-Wallis rank-sum test for ASM rank differences between the annotated genic regions. This non-parametric method tests whether multiple samples were drawn from the same distribution and is the multivariate extension of the better-known Wilcoxon rank-sum test. This test allowed us to avoid selecting a specific threshold for ASM scores and does not assume a normal distribution of residuals. Of the 220,449 informative probes, 219,921 could be annotated to specific defined genic regions. Annotations were based on the *Homo sapiens* hg19 build from UCSC using the AnnotationHub Bioconductor package^47^ classifying probes as residing in introns (n = 100,254), 5′UTRs (n = 347), 3′UTRs (n = 2,341), coding regions (n = 2,569), intergenic regions (n = 110,186) and promoters (n = 4,224). A Nemenyi test for pairwise multiple comparisons of mean rank sums as implemented in the PMCMR R package^48^ was used for post-hoc comparisons. ENCODE tracks for DHS peaks in frontal cortex, frontal cerebrum, cerebellum, CD14+ monocytes, naïve B cells, H1 human embryonic stem cells (H1-hesc), heart, and fibroblasts were obtained from the UCSC genome browser (http://hgdownload.cse.ucsc.edu/goldenPath/hg19/encodeDCC/wgEncodeOpenChromDnase/). For the DHS enrichment analyses we tested whether the rank-sums for ASM scores differed significantly in informative probes defined by the presence or absence of DHS peaks using a Wilcoxon rank-sum test. Informative probes were ranked according to ASM scores with higher absolute ASM scores corresponding to lower ranks.

### Clonal bisulfite sequencing

Two regions were subsequently selected for clonal bisulfite sequencing analysis to further verify our findings and determine the precise allele-specific patterns of DNA methylation. Following sodium bisulfite treatment and bisulfite-PCR amplification, amplicons were cloned using the TOPO TA cloning method (Invitrogen, Paisley, UK) and sequenced with BigDye v1.1 sequencing chemistry (Applied Biosystems) (Table S20). Sequencing traces were visualized, quality controlled, and aligned using BiQ Analyzer.^49^ All data were tested for complete sodium bisulfite conversion, with an overall conversion rate > 99.9% estimated by BiQ Analyzer.

### Validation of ASM on the llumina 450K HumanMethylation microarray

Further analysis of ASM sites was undertaken on a larger collection of post-mortem brain samples (n = 34), comprising BA9, BA21, BA28/34, and cerebellum, which were also free of any neuropathology and neuropsychiatric disease. Additionally we analyzed matched pre-mortem whole blood samples, which were available for a subset (n = 8), as well as 5 unmatched blood samples (see Table S18). The 3 individuals profiled by MSNP were included in this analysis. DNA (500 ng) from each sample was treated with sodium bisulfite in duplicate, using the EZ-96 DNA methylation kit (Zymo Research, CA, USA). DNA methylation was quantified using the Illumina Infinium HumanMethylation450 BeadChip (Illumina Inc., CA, USA) run on an Illumina HiScan System (Illumina) using the manufacturers' standard protocol, with pre-processing and stringent quality control performed as previously described.^50^ We used the GenomicRanges package^51^ to extract data for all CpG sites within 1 kb of candidate ASM SNPs and examined patterns of DNA methylation across the 3 tissues. Intermediate DNA methylation was defined as an average methylation value between 0.4 and 0.6 across all individuals. To test for statistical significance of enrichment in intermediately methylated probes we used a hypergeometric distribution based on the number of probes tested and the background of intermediately methylated probes across the whole array. Annotation of genes in the methylation plots (**Fig. 5** and **Fig. 7B**) was obtained from the UCSC Genome Browser hg19 assembly. Imprinting control region (ICR) annotation was obtained from the web resource on human DMRs provided by the Department of Medical and Molecular Genetics, Kings College London (https://atlas.genetics.kcl.ac.uk) and lifted over from hg18 to hg19.

## Supplementary Material

KEPI_A_1127479_s03.docx

KEPI_A_1127479_s02.docx
