# Supplementary material for: Tissue-specific patterns of allelically-skewed DNA methylation
Source: Epigenetics. 2016 Jan 19;11(1):24–35. doi: 10.1080/15592294.2015.1127479 (PMC4846124; doi:10.1080/15592294.2015.1127479)
Supplement: KEPI_A_1127479_s03.docx [file kepi-11-01-1127479-s001.docx]

A B

C D

**Supplementary Figure 1. The overall distribution of ASM score is similar across brain regions and whole blood samples.** The distribution of ASM scores **(A)** and absolute ASM scores **(B)** is similar across cortex (BA9), cerebellum and whole blood. However, histograms of absolute ASM score using a threshold of ≥ 0.10 **(C)** and ≥ 0.20 **(D)** indicate a large number of loci characterized by high allelic-skewing of DNA methylation in cerebellum and whole blood compared to cortex (BA9).


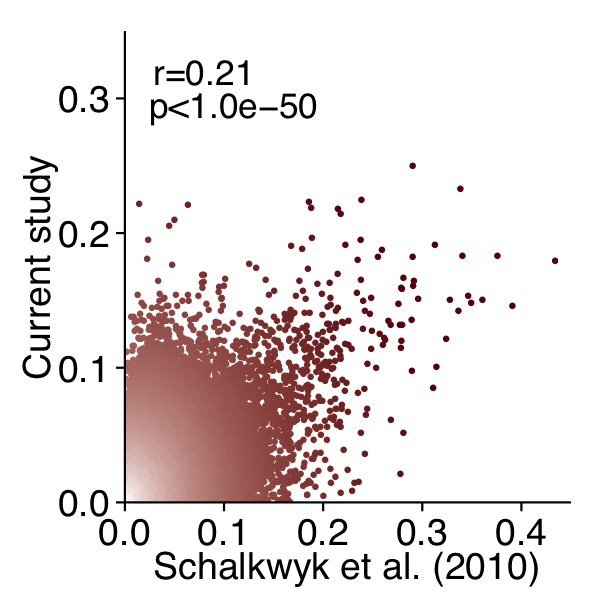


**Supplementary Figure 2. ASM scores in whole blood overlap with those identified in a previous study.** Across probes informative in both studies there is a significant correlation between whole blood ASM scores identified in the current study and previously by Schalkwyk *et al* (2010) (*r* = 0.21, *P* < 1.0 × 10^-50^). 9 of the 15 top-ranked blood ASM sites in our current study (**Table 1**) were informative in our previous dataset; 7 of these were characterized by an absolute ASM score ≥ 0.10.


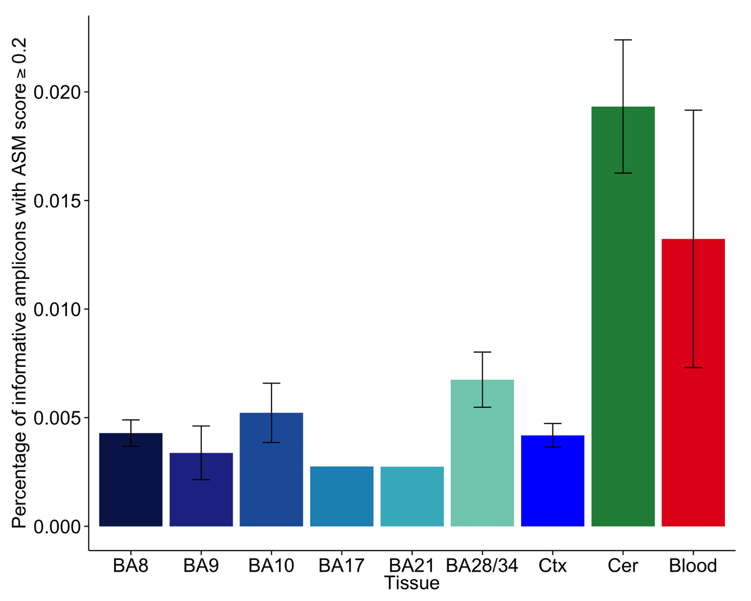


**Supplementary Figure 3. Large allelic asymmetries in DNA methylation are more prevalent in cerebellum and whole blood than cortex.** The average percentage of informative amplicons showing an ASM score ≥ 0.20 for the six cortical regions profiled as well as averaged across the cortical areas (Ctx) is consistently lower than in cerebellum (Cer) or blood. Standard errors are shown for tissues for which samples were available from all three individuals.


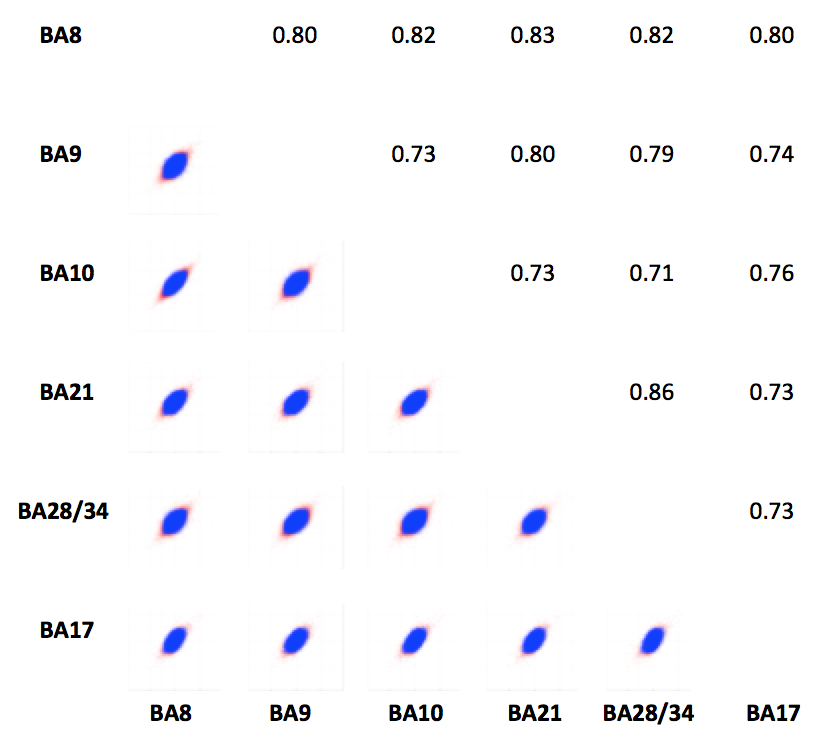


**Supplementary Figure 4. ASM scores are highly correlated across cortical regions.**

Lower triangular matrix: scatterplots of ASM scores, with probes characterized by absolute ASM scores ≥ 0.10 colored in red. Upper triangular matrix: correlations of ASM scores filtered for loci with at least one amplicon showing an absolute ASM score ≥ 0.10.

**A B**

**C D**

**Supplementary Figure 5. Informative probes annotated to genic promoter regions are enriched for elevated ASM scores.** A Kruskal-Wallis non-parametric test showed a significantly different distribution of ranked ASM scores between coding, 5’UTR, intergenic, intronic, promoter and 3’ UTR regions in informative cortex, cerebellum, blood and cross-tissue MSNP probes (*P* range = 2.21 × 10^26^ - 2.06 × 10^12^). Post-hoc analysis showed that the differences were primarily driven by an enrichment of elevated ASM scores in annotated promoter regions for **(A)** cortex (BA9), **(B)** cerebellum, **(C)** whole blood and **(D)** cross-tissue ASM scores. The mean number of informative probes per bin is displayed as a grey horizontal line.

**A**

**B**

**C**

**Supplementary Figure 6. DNase I hypersensitive (DHS) sites are enriched for loci characterized by high ASM scores.** DHS peaks overlapping informative MSNP probes are enriched for elevated ASM scores (see **Supplementary Table 3**). The histograms show the distribution of ASM score ranks for informative probes residing in ENCODE DHS peaks from frontal cortex **(A)**, cerebellum **(B)** and monocyte-CD14+ cells **(C)**. Shown is the distribution of ASM score ranks identified in cortex (BA9) (blue, top left), cerebellum (green, top right), whole blood (red, bottom left) and in the cross-tissue analysis (black, bottom right) across informative MSNP probes within ENCODE DHS peaks. All histograms show a clear peak in lower ranks with a uniform distribution across the rest of the rank spectrum, illustrating the higher than expected prevalence of probes with high ASM scores in the informative DHS regions.


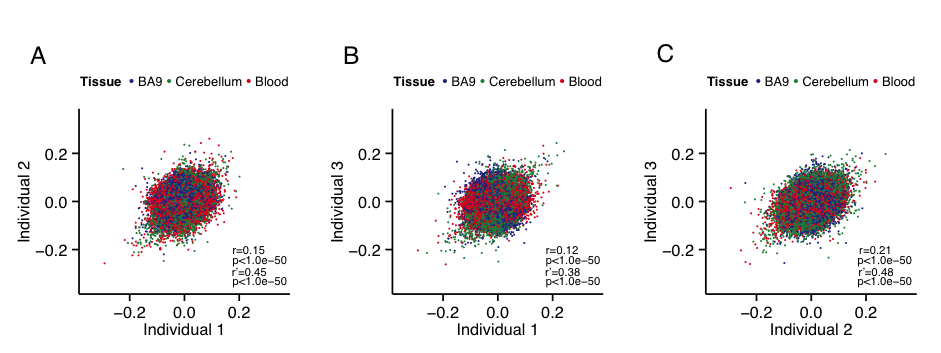


**Supplementary Figure 7. Individuals are more highly correlated for loci characterized by high ASM scores.** ASM scores are significantly correlated between individuals, particularly at loci with an ASM score ≥ 0.10 for at least one of the two compared individuals (subset correlation r’).  **(A)-(C)** Scatterplots of ASM scores across individuals plotted for each informative probe in cortex (BA9), cerebellum and whole blood.

A


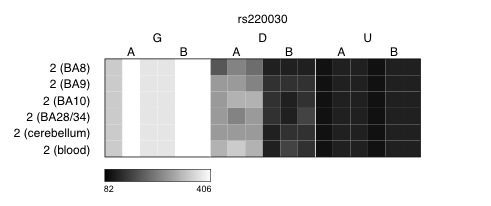


B


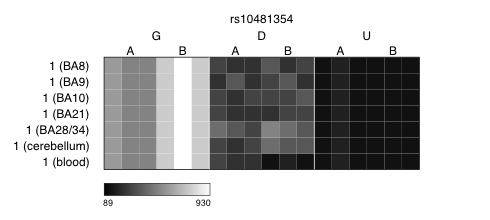


C
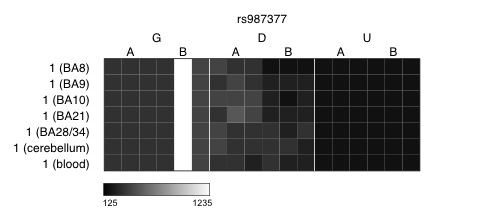


D
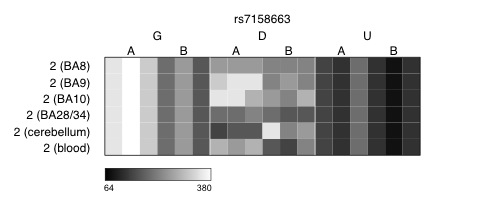


E
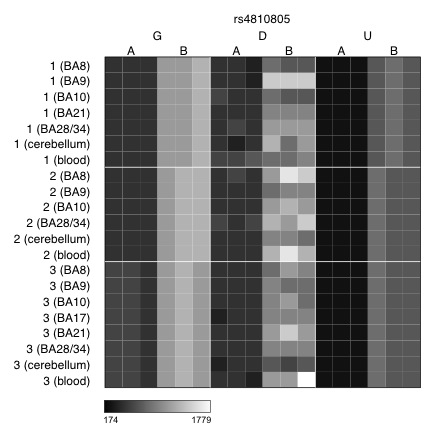


F
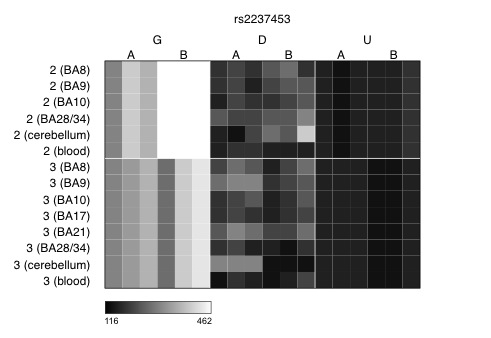


G
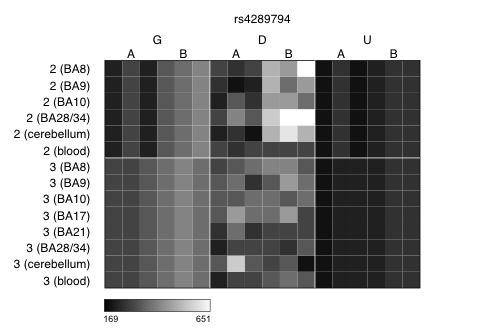


H
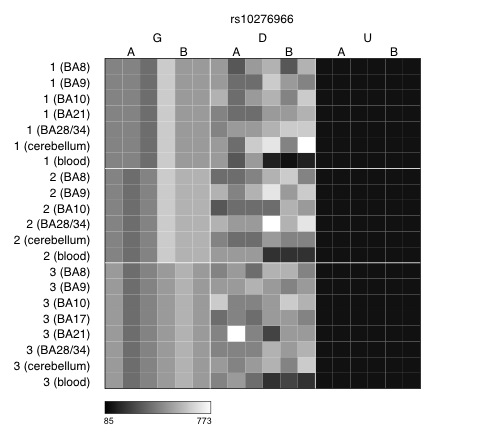


I
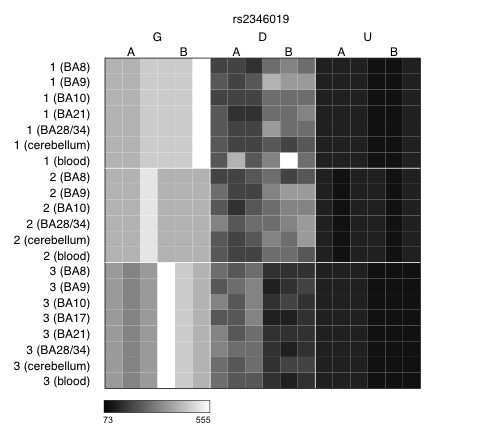


**Supplementary Figure 8. Several ASM sites were identified in known or suspected imprinted regions.** Heatmaps display allele signal intensities for genomic DNA (G), MSRE-digested DNA (D) and fully unmethylated, MSRE-digested DNA (U) in all tissues. A and B denote the two alleles of the SNP and brightness represents the quantile normalized signal intensity, with the scale displayed below the heatmap. Shown are data from probes in the vicinity of known imprinted genes: **(A)** rs220030 (*SNRPN*), **(B)** rs10481354 (*DLGAP2*), **(C)** rs987377 (*AIM1*), **(D)** rs7158663 (*MEG3*), **(E)** rs4810805 (*BLCAP*) and **(F)** rs2237453 (*GRB10*). Also shown are data from probes in the vicinity of suspected imprinted loci: **(G)** rs4289794 (*TRAPPC9*), **(H)** rs10276966 (*EVX1*), **(I)** rs2346019 (*VTRNA2-1*).


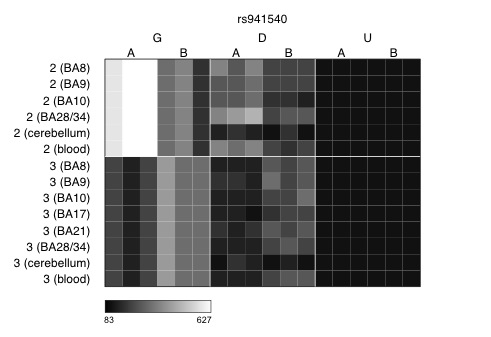


**Supplementary Figure 9. Some sites showing allelic-flipping in ASM are not located near known or suspected imprinted regions.** The heatmap displays allele signal intensities for genomic DNA (G), MSRE-digested DNA (D) and fully unmethylated, MSRE-digested DNA (U) in all tissues. A and B denote the two alleles of the SNP and brightness represents the quantile normalized signal intensity, with the scale displayed below the heatmap. The heatmap shows rs941540 (*ITPKI*), which is located in a region not previously shown to be genomically imprinted.
