# Supplementary material for: Tissue-specific patterns of allelically-skewed DNA methylation
Source: Epigenetics. 2016 Jan 19;11(1):24–35. doi: 10.1080/15592294.2015.1127479 (PMC4846124; doi:10.1080/15592294.2015.1127479)
Supplement: KEPI_A_1127479_s02.docx [file kepi-11-01-1127479-s002.docx]

| **Supplementary Table 1. Average absolute ASM scores for each sample.** | | | | | | | | | |
| --- | --- | --- | --- | --- | --- | --- | --- | --- | --- |
|  | **BA8** | **BA9** | **BA10** | **BA17** | **BA21** | **BA28/34** | **Cerebellum** | **Blood** | **Average** |
| Individual 1 | 0.026 | 0.025 | 0.028 |  | 0.023 | 0.027 | 0.023 | 0.028 | 0.026 |
| Individual 2 | 0.023 | 0.027 | 0.024 |  |  | 0.026 | 0.028 | 0.026 | 0.025 |
| Individual 3 | 0.025 | 0.027 | 0.025 | 0.024 | 0.023 | 0.023 | 0.030 | 0.024 | 0.025 |
| Average | 0.025 | 0.026 | 0.026 | 0.024 | 0.023 | 0.025 | 0.027 | 0.026 | 0.025 |

| **Supplementary Table 2. Percentage of amplicons characterized by an ASM score** ≥ **0.10 for each sample.** | | | | | | | | | |
| --- | --- | --- | --- | --- | --- | --- | --- | --- | --- |
|  | **BA8** | **BA9** | **BA10** | **BA17** | **BA21** | **BA28/34** | **Cerebellum** | **Blood** | **Average** |
| Individual 1 | 0.68% | 0.40% | 0.84% |  | 0.37% | 0.80% | 0.56% | 0.97% | 0.66% |
| Individual 2 | 0.42% | 0.64% | 0.44% |  |  | 0.64% | 1.36% | 1.03% | 0.76% |
| Individual 3 | 0.47% | 0.73% | 0.55% | 0.43% | 0.31% | 0.34% | 1.50% | 0.51% | 0.60% |
| Average | 0.52% | 0.59% | 0.61% | 0.43% | 0.34% | 0.59% | 1.14% | 0.84% | 0.63% |

| **Supplementary Table 3. Enrichment of high ASM scores in DNase I hypersensitivity peaks spanning informative MSNP probes.** | | | | | |
| --- | --- | --- | --- | --- | --- |
| Tissue/cell dataset from ENCODE | Number of informative probes in DHS peaks | Enrichment *P* value^*^ | | | |
|  |  | BA9 ASM score | Cerebellum ASM score | Blood ASM score | Cross-tissue ASM score |
| Frontal cortex | 7746 | 4.59 ×10^-38^ | 5.69 ×10^-58^ | 1.41 ×10^-43^ | 1.19 ×10^-81^ |
| Cerebrum frontal | 8294 | 8.84 ×10^-51^ | 1.75 ×10^-80^ | 3.19 ×10^-42^ | 2.92 ×10^-107^ |
| Cerebellum | 7228 | 2.10 ×10^-47^ | 3.51 ×10^-220^ | 1.09 ×10^-27^ | 4.74 ×10^-181^ |
| Monocytes - CD14+ | 8316 | 4.98 ×10^-15^ | 4.13 ×10^-24^ | 7.41 ×10^-52^ | 5.43 ×10^-53^ |
| Naïve B cell | 3908 | 4.71 ×10^-31^ | 2.10 ×10^-37^ | 2.99 ×10^-96^ | 1.35 ×10^-99^ |
| Heart | 7268 | 5.05 ×10^-42^ | 1.19 ×10^-54^ | 1.78 ×10^-55^ | 3.86 ×10^-93^ |
| Fibroblast | 4316 | 7.50 ×10^-25^ | 1.67 ×10^-24^ | 1.85 ×10^-20^ | 2.07 ×10^-42^ |
| H1-hesc | 7208 | 2.92 ×10^-44^ | 8.77 ×10^-67^ | 3.52 ×10^-56^ | 1.98 ×10^-93^ |
| * *P* values are based on a Wilcoxon rank-sum test comparing ASM ranks in DHS and non-DHS informative probes | | | | | |

| **Supplementary Table 4. Correlations of ASM scores between individuals.** | | | | | | | | |
| --- | --- | --- | --- | --- | --- | --- | --- | --- |
|  | **Correlation (*r*)** | | |  | **Subset correlation (*r’*)^*^** | | |  |
|  | **BA9** | **Cerebellum** | **Blood** | **All tissues^+^** | **BA9** | **Cerebellum** | **Blood** | **All tissues^+^** |
| Individual 1 vs. 2 | 0.15 | 0.19 | 0.12 | 0.15 | 0.45 | 0.51 | 0.41 | 0.45 |
| Individual 1 vs. 3 | -0.01 | 0.23 | 0.14 | 0.12 | 0.12 | 0.55 | 0.41 | 0.38 |
| Individual 2 vs. 3 | 0.15 | 0.28 | 0.17 | 0.21 | 0.34 | 0.52 | 0.53 | 0.48 |
| ^*^ The subset correlation *r’* is calculated using only probes for which at least one of the two compared individuals exhibit an ASM score ≥ 0.10 in the given tissue.  ^+^ Cerebellum, cortex (BA9) and whole blood | | | | | | | | |

| **Supplementary Table 5. Top 20 variable ASM sites in whole blood, defined by range of ASM scores.** | | | | | | | | | |
| --- | --- | --- | --- | --- | --- | --- | --- | --- | --- |
| **Rank** | **SNP ID** | **Location** | **Associated gene (s)** | **Blood ASM scores** | | | **Range per tissue** | | |
|  |  |  |  | **Ind 1** | **Ind 2** | **Ind 3** | **Blood** | **Cerebellum** | **BA9** |
| 1 | SNP_A-8579417 (rs1538116) | 1q24.1 | *MGST3, LOC400794* | - | -0.29 | 0.06 | 0.35 | 0.24 | 0.32 |
| 2 | SNP_A-8312502 (rs17469830) | 14q12 | *ARHGAP5, NUBPL, C14orf128* | -0.07 | 0.21 | - | 0.28 | 0.12 | 0.03 |
| 3 | SNP_A-1802351 (rs509062) | 20q13.32 | *PPP4R1L* | -0.16 | - | 0.08 | 0.25 | 0.13 | 0.05 |
| 4 | SNP_A-2019421 (rs2244352) | 21q22.2 | *WRB* | -0.19 | 0.05 | - | 0.24 | 0.36 | 0.29 |
| 5 | SNP_A-8324660 (rs6438837) | 3q21.2 | *KALRN* | -0.14 | 0.09 | -0.06 | 0.24 | 0.16 | 0.10 |
| 6 | SNP_A-4232094 (rs8179356) | 1p21.2 | *FRRS1* | - | 0.08 | -0.16 | 0.23 | 0.11 | 0.07 |
| 7 | SNP_A-1855770 (rs3764124) | 13q34 | *CUL4A* | -0.11 | 0.12 | 0.06 | 0.23 | 0.16 | 0.15 |
| 8 | SNP_A-4219174 (rs2346019) | 5q31.1 | *TGFBI, VTRNA2*^b^ | 0.04 | 0.07 | -0.16 | 0.23 | 0.23 | 0.30 |
| 9 | SNP_A-4208914 (rs927651) | 20q13.2 | *CYP24A1* | 0.15 | -0.08 | 0.00 | 0.23 | 0.25 | 0.17 |
| 10 | SNP_A-2275375 (rs7694398) | 4p16.1 | *CNO, KIAA0232* | 0.12 | -0.10 | -0.01 | 0.22 | 0.08 | 0.05 |
| 11 | SNP_A-1910565 (rs1040762) | 20p11.23 | *RIN2, SLC24A3* | 0.01 | -0.20 |  | 0.21 | 0.04 | 0.09 |
| 12 | SNP_A-8448479 (rs12837024) | Xq22.3 | *CLDN2, RIPPLY1* | -0.06 | - | 0.15 | 0.21 | 0.10 | 0.15 |
| 13 | SNP_A-2074691 (rs7065328) | Xq13.1 | *TEX11* | -0.10 | - | 0.10 | 0.21 | 0.08 | 0.01 |
| 14 | SNP_A-8330843 (rs896040) | 18q23 | *GALR1, MBP* | 0.14 | -0.05 | -0.06 | 0.21 | 0.11 | 0.06 |
| 15 | SNP_A-8605034 (rs17799404) | 4q13.2 | *CENPC1, LOC100144602* | 0.13 | - | -0.07 | 0.20 | 0.05 | 0.01 |
| 16 | SNP_A-8633376 (rs318657) | 5p15.33 | *LOC285577, C5orf38* | -0.11 | 0.09 | - | 0.20 | 0.11 | 0.08 |
| 17 | SNP_A-4239614 (rs1890070) | 1q41 | *HHIPL2, DUSP10* | -0.16 | 0.04 | - | 0.20 | 0.02 | 0.02 |
| 18 | SNP_A-2256850 (rs1164193) | 3q13.13 | *PVRL3, FLJ25363* | 0.08 | -0.12 | - | 0.20 | 0.01 | 0.08 |
| 19 | SNP_A-1869018 (rs1984713) | 4p12 | *CNGA1* | - | -0.18 | 0.02 | 0.20 | 0.15 | 0.14 |
| 20 | SNP_A-8436908 (rs4810805) | 20q11.23 | *CTNNBL1, BLCAP*^a^ | -0.14 | 0.05 | 0.06 | 0.20 | 0.05 | 0.07 |
| ^a^ Known imprinted gene  ^b^ Suspected imprinted gene | | | | | | | | | |

| **Supplementary Table 6. Top 20 variable ASM sites in cerebellum, defined by range of ASM scores.** | | | | | | | | | |
| --- | --- | --- | --- | --- | --- | --- | --- | --- | --- |
| **Rank** | **SNP ID** | **Location** | **Associated gene (s)** | **Cerebellum ASM scores** | | | | **Range per tissue** | |
|  |  |  |  | **Ind 1** | **Ind 2** | **Ind 3** | **Cerebellum** | **Blood** | **BA9** |
| 1 | SNP_A-2019421 (rs2244352) | 21q22.2 | *WRB* | -0.22 | 0.14 | - | 0.36 | 0.24 | 0.29 |
| 2 | SNP_A-8492800 (rs2237453) | 7p12.1 | *GRB10*^a^ | - | 0.06 | -0.23 | 0.30 | 0.11 | 0.08 |
| 3 | SNP_A-8291694 (rs941540) | 14q32.12 | *ITPK1* | - | 0.10 | -0.17 | 0.27 | 0.07 | 0.13 |
| 4 | SNP_A-8717059 (rs12713666) | 2p13.3 | *ARHGAP25* | -0.08 | 0.18 | 0.04 | 0.26 | 0.15 | 0.21 |
| 5 | SNP_A-4208914 (rs927651) | 20q13.2 | *CYP24A1* | 0.08 | -0.17 | -0.06 | 0.25 | 0.23 | 0.17 |
| 6 | SNP_A-8596407 (rs10778439) | 12q23.3 | *NUAK1, C12orf75* | 0.00 | - | -0.25 | 0.25 | 0.03 | 0.09 |
| 7 | SNP_A-1788232 (rs11854691) | 15q11.2 | *C15orf2*^a^*,* *PWRN2, NDN*^a^ | -0.15 | 0.10 | - | 0.25 | 0.06 | 0.17 |
| 8 | SNP_A-2118217 (rs1695824) | 1p36.33 | *VWA1, TMEM88B* | -0.22 | - | 0.03 | 0.25 | 0.16 | 0.13 |
| 9 | SNP_A-2252209 (rs940596) | 15q11.2 | *C15orf2*^a^*, PWRN2, NDN*^a^ | -0.10 | 0.11 | -0.13 | 0.25 | 0.04 | 0.09 |
| 10 | SNP_A-8713358 (rs16890883) | 4p15.33 | *CPEB2, LOC152742* | 0.04 | -0.21 | 0.01 | 0.24 | 0.14 | 0.17 |
| 11 | SNP_A-8397290 (rs4289794) | 8q24.3 | *TRAPPC9*^b^ | - | 0.10 | -0.14 | 0.24 | 0.09 | 0.08 |
| 12 | SNP_A-8579417 (rs1538116) | 1q24.1 | *MGST3, LOC400794* | - | -0.24 | 0.00 | 0.24 | 0.35 | 0.32 |
| 13 | SNP_A-8321422 (rs3759917) | 15q26.1 | *ST8SIA2, SLCO3A1* | 0.00 | 0.24 | - | 0.24 | 0.12 | 0.14 |
| 14 | SNP_A-1871907 (rs9460408) | 6p22.3 | *ID4, MIR548A1* | - | 0.17 | -0.06 | 0.23 | 0.12 | 0.08 |
| 15 | SNP_A-4219174 (rs2346019) | 5q31.1 | *TGFB, VTRNA2* | 0.00 | 0.10 | -0.13 | 0.23 | 0.23 | 0.30 |
| 16 | SNP_A-8353238 (rs9570315) | 13q21.2 | *MIR3169, TDRD3* | -0.07 | - | 0.17 | 0.23 | 0.16 | 0.09 |
| 17 | SNP_A-1880775 (rs6116750) | 20p12.3 | *PROKR2, LOC643406* | - | 0.12 | -0.11 | 0.23 | 0.13 | 0.18 |
| 18 | SNP_A-2244974 (rs941483) | 14q32.12 | *ASB2* | 0.01 | -0.17 | 0.05 | 0.23 | 0.04 | 0.05 |
| 19 | SNP_A-8638465 (rs11633486) | 15q11.2 | *C15orf2*^a^*, PWRN2, NDN*^a^ | -0.13 | 0.10 | - | 0.23 | 0.11 | 0.16 |
| 20 | SNP_A-8437947 (rs2858602) | 22q13.33 | *C22orf34, FAM19A5* | -0.07 | 0.11 | 0.16 | 0.22 | 0.06 | 0.15 |
| ^a^ Known imprinted gene  ^b^ Suspected imprinted gene | | | | | | | | | |

| **Supplementary Table 7. Top 20 variable ASM sites in cortex (BA9), as defined by range of ASM scores.** | | | | | | | | | | |
| --- | --- | --- | --- | --- | --- | --- | --- | --- | --- | --- |
| **Rank** | **SNP ID** | **Location** | **Associated gene (s)** | **BA9 ASM scores** | | | | **Range per tissue** | | |
|  |  |  |  | **Ind 1** | **Ind 2** | **Ind 3** | **BA9** | | **Cerebellum** | **Blood** |
| 1 | SNP_A-8579417 (rs1538116) | 1q24.1 | *MGST3, LOC400794* | - | -0.24 | 0.08 | 0.32 | | 0.24 | 0.35 |
| 2 | SNP_A-8424056 (rs3922835) | 18q12.1 | *CDH2*^b^, *CHST9* | -0.15 | - | 0.17 | 0.32 | | 0.18 | 0.04 |
| 3 | SNP_A-4219174 (rs2346019) | 5q31.1 | *TGFBI, VTRNA2* | 0.10 | 0.13 | -0.17 | 0.30 | | 0.23 | 0.23 |
| 4 | SNP_A-2019421 (rs2244352) | 21q22.2 | *WRB* | -0.15 | 0.14 | - | 0.29 | | 0.36 | 0.24 |
| 5 | SNP_A-8448997 (rs17346747) | 8p23.2 | *CSMD1* | -0.11 | 0.01 | 0.15 | 0.26 | | 0.10 | 0.03 |
| 6 | SNP_A-8692937 (rs4605656) | 4q24 | *CXXC4, TACR3* | -0.19 | - | 0.06 | 0.25 | | 0.16 | 0.18 |
| 7 | SNP_A-8658815 (rs9807659) | 18q11.2 | *LAMA3* | -0.08 | 0.02 | 0.17 | 0.24 | | 0.12 | 0.10 |
| 8 | SNP_A-8311269 (rs403594) | 1p32.3 | *ACOT11* | -0.12 | 0.03 | 0.12 | 0.24 | | 0.11 | 0.02 |
| 9 | SNP_A-8634251 (rs1209228) | 14q24.2 | *RGS6, SIPA1L1* | -0.18 | 0.06 | 0.01 | 0.24 | | 0.19 | 0.15 |
| 10 | SNP_A-1951087 (rs9357087) | 6p24.2 | *NEDD9* | - | 0.03 | -0.19 | 0.23 | | 0.07 | 0.07 |
| 11 | SNP_A-8487535 (rs503755) | 2q21.3 | *TMEM163* | - | 0.10 | -0.12 | 0.22 | | 0.01 | 0.09 |
| 12 | SNP_A-8329713 (rs16825906) | 3q13.31 | *LSAMP, IGSF11, LOC285194* | -0.04 | 0.04 | 0.19 | 0.22 | | 0.21 | 0.13 |
| 13 | SNP_A-8301613 (rs4380126) | 18q23 | *C18orf62, TSHZ1* | 0.10 | -0.02 | -0.12 | 0.22 | | 0.17 | 0.10 |
| 14 | SNP_A-8368457 (rs7307323) | 12p11.22 | *FAR2, CCDC91* | - | 0.19 | -0.03 | 0.21 | | 0.09 | 0.05 |
| 15 | SNP_A-8399845 (rs197493) | 6q24.2 | *HIVEP2 , LOC153910* | -0.08 | -0.02 | 0.13 | 0.21 | | 0.15 | 0.03 |
| 16 | SNP_A-8717059 (rs12713666) | 2p13.3 | *ARHGAP25* | -0.13 | 0.08 | 0.02 | 0.21 | | 0.26 | 0.15 |
| 17 | SNP_A-8515165 (rs7892015) | Xq21.1 | *POU3F4, SH3BGRL* | -0.03 | - | 0.18 | 0.21 | | 0.15 | 0.10 |
| 18 | SNP_A-8613922 (rs1009800) | 13q21.33 | *KLHL1, PCDH9* | -0.06 | 0.03 | 0.16 | 0.21 | | 0.11 | 0.05 |
| 19 | SNP_A-8482424 (rs10880337) | 12q12 | *ADAMTS20, PRICKLE1* | -0.11 | 0.10 | 0.05 | 0.21 | | 0.15 | 0.08 |
| 20 | SNP_A-2246435 (rs7070457) | 10q21.1 | *ZWINT* | 0.12 | - | -0.09 | 0.20 | | 0.19 | 0.13 |
| ^b^ Suspected imprinted gene | | | | | | | | | | |

| **Supplementary Table 8. Illumina 450K probes within 1kb of 100 top-ranked ASM sites.** | | |
| --- | --- | --- |
| **Top 100 ASM sites** | **SNPs** | **CpG sites** |
| Blood ASM | 27 | 102 |
| Cerebellum ASM | 20 | 87 |
| BA9 ASM | 40 | 143 |
| Cross-tissue ASM | 34 | 148 |
| Tissue-specific ASM | 22 | 50 |
| Variable ASM | 22 | 65 |
| Variable ASM (blood) | 19 | 58 |
| Variable ASM (cerebellum) | 18 | 59 |
| Variable ASM (BA9) | 20 | 56 |

| **Supplementary Table 9. Illumina 450K probes within 1kb of the top 100 ASM SNPs in whole blood.** | | | | | | | |
| --- | --- | --- | --- | --- | --- | --- | --- |
| **Rank** | **ASM in blood** | **SNP** | **Chr** | **Mapinfo SNP** | **CpG site** | **Mapinfo CpG** | **Distance** |
| 3 | 0.23 | rs10234308 | 7 | 22528590 | cg10016647 | 22528033 | 557 |
| 4 | 0.23 | rs927000 | 20 | 43598705 | cg17104026 | 43598376 | 329 |
| 10 | 0.22 | rs4687210 | 3 | 191046770 | cg25342125 | 191046380 | 390 |
|  |  |  |  |  | cg19066520 | 191046547 | 223 |
|  |  |  |  |  | cg23528751 | 191046721 | 49 |
|  |  |  |  |  | cg00473167 | 191046872 | 102 |
|  |  |  |  |  | cg17596905 | 191046949 | 179 |
|  |  |  |  |  | cg23817170 | 191046997 | 227 |
|  |  |  |  |  | cg09688588 | 191047460 | 690 |
|  |  |  |  |  | cg14972328 | 191046866 | 96 |
|  |  |  |  |  | cg26325444 | 191047058 | 288 |
| 12 | 0.22 | rs12978286 | 19 | 17861868 | cg22317846 | 17862385 | 517 |
|  |  |  |  |  | cg02639359 | 17862017 | 149 |
|  |  |  |  |  | cg26307105 | 17861017 | 851 |
|  |  |  |  |  | cg08206267 | 17862104 | 236 |
|  |  |  |  |  | cg11737334 | 17862210 | 342 |
|  |  |  |  |  | cg24783510 | 17862150 | 282 |
| 13 | 0.22 | rs10481354 | 8 | 1704030 | cg19131313 | 1704013 | 17 |
|  |  |  |  |  | cg17159058 | 1704075 | 45 |
| 14 | 0.21 | rs1542180 | 2 | 177028767 | cg12634591 | 177028606 | 161 |
|  |  |  |  |  | cg06145336 | 177027931 | 836 |
|  |  |  |  |  | cg10304824 | 177028804 | 37 |
|  |  |  |  |  | cg13316854 | 177027856 | 911 |
|  |  |  |  |  | cg09387749 | 177028680 | 87 |
|  |  |  |  |  | cg25938806 | 177028162 | 605 |
|  |  |  |  |  | cg01128482 | 177028621 | 146 |
|  |  |  |  |  | cg00005847 | 177029073 | 306 |
|  |  |  |  |  | cg24541426 | 177029459 | 692 |
|  |  |  |  |  | cg23981871 | 177028714 | 53 |
|  |  |  |  |  | cg05021643 | 177029608 | 841 |
| 25 | 0.20 | rs17312325 | X | 68442113 | cg00427681 | 68441389 | 724 |
|  |  |  |  |  | cg03350825 | 68442327 | 214 |
|  |  |  |  |  | cg22337892 | 68441690 | 423 |
| 27 | 0.20 | rs1024611 | 17 | 32579788 | cg13648045 | 32579022 | 766 |
| 30 | 0.20 | rs12493005 | 3 | 176912899 | cg03356026 | 176912588 | 311 |
| 37 | 0.19 | rs12510921 | 4 | 185189278 | cg04960065 | 185189300 | 22 |
| 43 | 0.19 | rs906805 | 2 | 28604879 | cg01273330 | 28605224 | 345 |
| 44 | 0.18 | rs220030 | 15 | 25199768 | cg21746532 | 25198793 | 975 |
|  |  |  |  |  | cg26875073 | 25200490 | 722 |
|  |  |  |  |  | cg02152271 | 25199270 | 498 |
|  |  |  |  |  | cg02125271 | 25200406 | 638 |
|  |  |  |  |  | cg03858387 | 25199164 | 604 |
|  |  |  |  |  | cg01432432 | 25199028 | 740 |
|  |  |  |  |  | cg08372135 | 25199057 | 711 |
|  |  |  |  |  | cg18506672 | 25200253 | 485 |
|  |  |  |  |  | cg12298755 | 25199713 | 55 |
| 48 | 0.18 | rs2252397 | 7 | 140225750 | cg01499591 | 140226367 | 617 |
| 50 | 0.18 | rs999556 | 5 | 150473674 | cg23290159 | 150474234 | 560 |
| 56 | 0.18 | rs9526072 | 13 | 32883583 | cg26941801 | 32882845 | 738 |
|  |  |  |  |  | cg11448807 | 32884114 | 531 |
| 57 | 0.18 | rs7010076 | 8 | 105342827 | cg13045555 | 105342365 | 462 |
|  |  |  |  |  | cg04554929 | 105342491 | 336 |
|  |  |  |  |  | cg15172053 | 105342214 | 613 |
|  |  |  |  |  | cg23108931 | 105342351 | 476 |
|  |  |  |  |  | cg17696517 | 105343133 | 306 |
| 58 | 0.18 | rs485219 | 8 | 70065359 | cg05959464 | 70064456 | 903 |
| 62 | 0.18 | rs7210715 | 17 | 7196390 | cg00901493 | 7197375 | 985 |
| 64 | 0.17 | rs17366322 | 2 | 11272784 | cg05517905 | 11273075 | 291 |
|  |  |  |  |  | cg09045196 | 11272424 | 360 |
|  |  |  |  |  | cg12453288 | 11273229 | 445 |
|  |  |  |  |  | cg10334385 | 11272868 | 84 |
|  |  |  |  |  | cg03251967 | 11273291 | 507 |
|  |  |  |  |  | cg15704155 | 11272399 | 385 |
|  |  |  |  |  | cg27641709 | 11272899 | 115 |
| 67 | 0.17 | rs16942894 | 12 | 114026006 | cg12492938 | 114025383 | 623 |
| 70 | 0.17 | rs9314433 | 8 | 1467648 | cg00598912 | 1468625 | 977 |
| 71 | 0.17 | rs2071780 | X | 47341821 | cg26176649 | 47342033 | 212 |
|  |  |  |  |  | cg02124059 | 47341898 | 77 |
|  |  |  |  |  | cg22238863 | 47342101 | 280 |
|  |  |  |  |  | cg22713892 | 47342160 | 339 |
|  |  |  |  |  | cg23534593 | 47342469 | 648 |
|  |  |  |  |  | cg21161328 | 47342498 | 677 |
|  |  |  |  |  | cg24559073 | 47342471 | 650 |
|  |  |  |  |  | cg19867709 | 47342478 | 657 |
|  |  |  |  |  | cg04302192 | 47341740 | 81 |
|  |  |  |  |  | cg04575501 | 47342663 | 842 |
|  |  |  |  |  | cg14388993 | 47342690 | 869 |
|  |  |  |  |  | cg01887353 | 47342480 | 659 |
| 78 | 0.17 | rs2332036 | 3 | 121714391 | cg20356878 | 121714668 | 277 |
|  |  |  |  |  | cg11854227 | 121714109 | 282 |
| 83 | 0.17 | rs4810485 | 20 | 44747947 | cg21601405 | 44747006 | 941 |
|  |  |  |  |  | cg16686951 | 44747351 | 596 |
| 86 | 0.16 | rs10883857 | 10 | 105127587 | cg23418095 | 105128462 | 875 |
|  |  |  |  |  | cg14125903 | 105126693 | 894 |
|  |  |  |  |  | cg00245789 | 105128183 | 596 |
|  |  |  |  |  | cg20188621 | 105127192 | 395 |
|  |  |  |  |  | cg11667101 | 105127581 | 6 |
|  |  |  |  |  | cg10301212 | 105126702 | 885 |
|  |  |  |  |  | cg26978691 | 105128013 | 426 |
|  |  |  |  |  | cg18732587 | 105127670 | 83 |
|  |  |  |  |  | cg25713684 | 105127687 | 100 |
|  |  |  |  |  | cg18586343 | 105127615 | 28 |
|  |  |  |  |  | cg20684973 | 105127632 | 45 |
|  |  |  |  |  | cg16500454 | 105127701 | 114 |
|  |  |  |  |  | cg01360325 | 105127811 | 224 |
| 88 | 0.16 | rs4075600 | 16 | 86929340 | cg07824497 | 86930033 | 693 |
|  |  |  |  |  | cg08569253 | 86930198 | 858 |
|  |  |  |  |  | cg05490489 | 86930336 | 996 |
| 90 | 0.16 | rs10411704 | 19 | 35800662 | cg22238209 | 35800743 | 81 |
|  |  |  |  |  | cg07918620 | 35800925 | 263 |
|  |  |  |  |  | cg15761414 | 35801014 | 352 |
|  |  |  |  |  | cg02776658 | 35800589 | 73 |

| **Supplementary Table 10. Illumina 450K probes within 1kb of the top 100 ASM SNPs in cerebellum** | | | | | | | | | | | | | | |  |
| --- | --- | --- | --- | --- | --- | --- | --- | --- | --- | --- | --- | --- | --- | --- | --- |
| **Rank** | **Cerebellum ASM score** | | **SNP** | | **Chr** | | **Mapinfo SNP** | | **CpG site** | | **Mapinfo CpG** | | **Distance** | |  |
| 2 | 0.29 | | rs1003533 | | 5 | | 131755651 | | cg03347934 | | 131754928 | | 723 | |  |
| 3 | 0.26 | | rs7959070 | | 12 | | 92805682 | | cg25400182 | | 92806045 | | 363 | |  |
| 4 | 0.25 | | rs10234308 | | 7 | | 22528590 | | cg10016647 | | 22528033 | | 557 | |  |
| 6 | 0.24 | | rs12493005 | | 3 | | 176912899 | | cg03356026 | | 176912588 | | 311 | |  |
| 7 | 0.24 | | rs1542180 | | 2 | | 177028767 | | cg13316854 | | 177027856 | | 911 | |  |
|  |  | |  | |  | |  | | cg06145336 | | 177027931 | | 836 | |  |
|  |  | |  | |  | |  | | cg25938806 | | 177028162 | | 605 | |  |
|  |  | |  | |  | |  | | cg12634591 | | 177028606 | | 161 | |  |
|  |  | |  | |  | |  | | cg01128482 | | 177028621 | | 146 | |  |
|  |  | |  | |  | |  | | cg09387749 | | 177028680 | | 87 | |  |
|  |  | |  | |  | |  | | cg23981871 | | 177028714 | | 53 | |  |
|  |  | |  | |  | |  | | cg10304824 | | 177028804 | | 37 | |  |
|  |  | |  | |  | |  | | cg00005847 | | 177029073 | | 306 | |  |
|  |  | |  | |  | |  | | cg24541426 | | 177029459 | | 692 | |  |
|  |  | |  | |  | |  | | cg05021643 | | 177029608 | | 841 | |  |
| 9 | 0.24 | | rs3098382 | | 5 | | 71462632 | | cg20954533 | | 71462729 | | 97 | |  |
| 12 | 0.23 | | rs7158663 | | 14 | | 101319424 | | cg19509303 | | 101318703 | | 721 | |  |
|  |  | |  | |  | |  | | cg07460524 | | 101319526 | | 102 | |  |
| 21 | 0.21 | | rs12413873 | | 10 | | 71212091 | | cg13358349 | | 71211210 | | 881 | |  |
|  |  | |  | |  | |  | | cg12712771 | | 71211212 | | 879 | |  |
|  |  | |  | |  | |  | | cg26465120 | | 71211268 | | 823 | |  |
|  |  | |  | |  | |  | | cg11279933 | | 71211301 | | 790 | |  |
|  |  | |  | |  | |  | | cg01566955 | | 71211468 | | 623 | |  |
|  |  | |  | |  | |  | | cg18382422 | | 71211551 | | 540 | |  |
|  |  | |  | |  | |  | | cg12610070 | | 71211762 | | 329 | |  |
|  |  | |  | |  | |  | | cg14388049 | | 71211838 | | 253 | |  |
| 24 | 0.21 | | rs2546890 | | 5 | | 158759900 | | cg07622001 | | 158758903 | | 997 | |  |
| 45 | 0.19 | | rs10861495 | | 12 | | 106142981 | | cg23539745 | | 106142108 | | 873 | |  |
| 63 | 0.18 | | rs971649 | | 8 | | 124732275 | | cg13177458 | | 124732088 | | 187 | |  |
| 65 | 0.18 | | rs7247675 | | 19 | | 28285519 | | cg17155612 | | 28284698 | | 821 | |  |
|  |  | |  | |  | |  | | cg02942594 | | 28284741 | | 778 | |  |
|  |  | |  | |  | |  | | cg03170611 | | 28284813 | | 706 | |  |
|  |  | |  | |  | |  | | cg22473312 | | 28284823 | | 696 | |  |
|  |  | |  | |  | |  | | cg08220598 | | 28284952 | | 567 | |  |
|  |  | |  | |  | |  | | cg17871739 | | 28284973 | | 546 | |  |
|  |  | |  | |  | |  | | cg24415565 | | 28284988 | | 531 | |  |
|  |  | |  | |  | |  | | cg25246092 | | 28285128 | | 391 | |  |
|  |  | |  | |  | |  | | cg03751272 | | 28285308 | | 211 | |  |
|  |  | |  | |  | |  | | cg06949053 | | 28285395 | | 124 | |  |
| 72 | 0.18 | | rs3762199 | | 20 | | 18487792 | | cg26234223 | | 18487771 | | 21 | |  |
|  |  | |  | |  | |  | | cg01866064 | | 18487951 | | 159 | |  |
|  |  | |  | |  | |  | | cg27634549 | | 18487976 | | 184 | |  |
|  |  | |  | |  | |  | | cg23490046 | | 18487999 | | 207 | |  |
|  |  | |  | |  | |  | | cg15613982 | | 18488017 | | 225 | |  |
|  |  | |  | |  | |  | | cg16156954 | | 18488094 | | 302 | |  |
|  |  | |  | |  | |  | | cg07973146 | | 18488185 | | 393 | |  |
|  |  | |  | |  | |  | | cg02081051 | | 18488312 | | 520 | |  |
|  |  | |  | |  | |  | | cg22243844 | | 18488366 | | 574 | |  |
|  |  | |  | |  | |  | | cg13313269 | | 18488622 | | 830 | |  |
| 73 | 0.18 | | rs220030 | | 15 | | 25199768 | | cg21746532 | | 25198793 | | 975 | |  |
|  |  | |  | |  | |  | | cg01432432 | | 25199028 | | 740 | |  |
|  |  | |  | |  | |  | | cg08372135 | | 25199057 | | 711 | |  |
|  |  | |  | |  | |  | | cg03858387 | | 25199164 | | 604 | |  |
|  |  | |  | |  | |  | | cg02152271 | | 25199270 | | 498 | |  |
|  |  | |  | |  | |  | | cg12298755 | | 25199713 | | 55 | |  |
|  |  | |  | |  | |  | | cg18506672 | | 25200253 | | 485 | |  |
|  |  | |  | |  | |  | | cg02125271 | | 25200406 | | 638 | |  |
|  |  | |  | |  | |  | | cg26875073 | | 25200490 | | 722 | |  |
| 76 | 0.18 | | rs2244352 | | 21 | | 40757973 | | cg01322405 | | 40757317 | | 656 | |  |
|  |  | |  | |  | |  | | cg00606841 | | 40757691 | | 282 | |  |
|  |  | |  | |  | |  | | cg22858667 | | 40757750 | | 223 | |  |
|  |  | |  | |  | |  | | cg26710963 | | 40757899 | | 74 | |  |
|  |  | |  | |  | |  | | cg09916765 | | 40758208 | | 235 | |  |
|  |  | |  | |  | |  | | cg21297395 | | 40758325 | | 352 | |  |
|  |  | |  | |  | |  | | cg21459460 | | 40758346 | | 373 | |  |
| 84 | 0.18 | | rs9469529 | | 6 | | 33587443 | | cg08355863 | | 33587496 | | 53 | |  |
|  |  | |  | |  | |  | | cg24603235 | | 33588219 | | 776 | |  |
|  |  | |  | |  | |  | | cg19889152 | | 33588278 | | 835 | |  |
|  |  | |  | |  | |  | | cg14639225 | | 33588302 | | 859 | |  |
| 85 | 0.18 | | rs1342331 | | 6 | | 144066159 | | cg06759993 | | 144065203 | | 956 | |  |
|  |  | |  | |  | |  | | cg08482167 | | 144066847 | | 688 | |  |
| 86 | 0.18 | | rs7185244 | | 16 | | 86546887 | | cg08142918 | | 86546374 | | 513 | |  |
|  |  | |  | |  | |  | | cg03697918 | | 86546628 | | 259 | |  |
|  |  | |  | |  | |  | | cg24908603 | | 86546631 | | 256 | |  |
|  |  | |  | |  | |  | | cg07056644 | | 86546785 | | 102 | |  |
|  |  | |  | |  | |  | | cg27453745 | | 86546938 | | 51 | |  |
|  |  | |  | |  | |  | | cg09338251 | | 86546979 | | 92 | |  |
|  |  | |  | |  | |  | | cg06834912 | | 86547203 | | 316 | |  |
|  |  | |  | |  | |  | | cg04787888 | | 86547322 | | 435 | |  |
|  |  | |  | |  | |  | | cg01243371 | | 86547386 | | 499 | |  |
|  |  | |  | |  | |  | | cg00551679 | | 86547530 | | 643 | |  |
|  |  | |  | |  | |  | | cg02783918 | | 86547544 | | 657 | |  |
| 87 | 0.18 | | rs12582221 | | 12 | | 125323240 | | cg17534092 | | 125322578 | | 662 | |  |
| 99 | 0.17 | | rs6713344 | | 2 | | 112814045 | | cg09071112 | | 112813174 | | 871 | |  |
|  |  | |  | |  | |  | | cg06723287 | | 112813613 | | 432 | |  |
|  |  | |  | |  | |  | | cg11931949 | | 112814041 | | 4 | |  |
|  |  | |  | |  | |  | | cg19917083 | | 112814452 | | 407 | |  |
| **Supplementary Table 11. CpG sites within 1kb of the top 100 ASM SNPs in BA9.** | | | | | | | | | | | | | | | |
| **Rank** | | **BA9 ASM score** | | **SNP** | | **Chr** | | **Mapinfo SNP** | | **CpG site** | | **Mapinfo CpG** | | **Distance** | |
| 1 | | 0.24 | | rs12493005 | | 3 | | 176912899 | | cg03356026 | | 176912588 | | 311 | |
| 3 | | 0.23 | | rs10234308 | | 7 | | 22528590 | | cg10016647 | | 22528033 | | 557 | |
| 7 | | 0.19 | | rs3098382 | | 5 | | 71462632 | | cg20954533 | | 71462729 | | 97 | |
| 9 | | 0.19 | | rs3121125 | | 1 | | 145382124 | | cg23685870 | | 145382340 | | 216 | |
|  | |  | |  | |  | |  | | cg07117663 | | 145382963 | | 839 | |
| 10 | | 0.19 | | rs1542180 | | 2 | | 177028767 | | cg13316854 | | 177027856 | | 911 | |
|  | |  | |  | |  | |  | | cg06145336 | | 177027931 | | 836 | |
|  | |  | |  | |  | |  | | cg25938806 | | 177028162 | | 605 | |
|  | |  | |  | |  | |  | | cg12634591 | | 177028606 | | 161 | |
|  | |  | |  | |  | |  | | cg01128482 | | 177028621 | | 146 | |
|  | |  | |  | |  | |  | | cg09387749 | | 177028680 | | 87 | |
|  | |  | |  | |  | |  | | cg23981871 | | 177028714 | | 53 | |
|  | |  | |  | |  | |  | | cg10304824 | | 177028804 | | 37 | |
|  | |  | |  | |  | |  | | cg00005847 | | 177029073 | | 306 | |
|  | |  | |  | |  | |  | | cg24541426 | | 177029459 | | 692 | |
|  | |  | |  | |  | |  | | cg05021643 | | 177029608 | | 841 | |
| 14 | | 0.18 | | rs17303015 | | 5 | | 43194137 | | cg10641714 | | 43193681 | | 456 | |
| 15 | | 0.18 | | rs4871852 | | 8 | | 23020396 | | cg23051664 | | 23020966 | | 570 | |
|  | |  | |  | |  | |  | | cg10964421 | | 23021093 | | 697 | |
|  | |  | |  | |  | |  | | cg01456571 | | 23021325 | | 929 | |
| 20 | | 0.18 | | rs6874600 | | 5 | | 73945738 | | cg20658167 | | 73944869 | | 869 | |
| 25 | | 0.18 | | rs7073504 | | 10 | | 60271987 | | cg07567376 | | 60271505 | | 482 | |
|  | |  | |  | |  | |  | | cg09696939 | | 60272079 | | 92 | |
|  | |  | |  | |  | |  | | cg07857251 | | 60272754 | | 767 | |
|  | |  | |  | |  | |  | | cg19940537 | | 60272938 | | 951 | |
| 29 | | 0.17 | | rs6929846 | | 6 | | 26458265 | | cg03553910 | | 26457928 | | 337 | |
|  | |  | |  | |  | |  | | cg00369351 | | 26458121 | | 144 | |
|  | |  | |  | |  | |  | | cg27318050 | | 26458189 | | 76 | |
|  | |  | |  | |  | |  | | cg22814929 | | 26458213 | | 52 | |
|  | |  | |  | |  | |  | | cg01542644 | | 26458326 | | 61 | |
|  | |  | |  | |  | |  | | cg11239695 | | 26458489 | | 224 | |
| 30 | | 0.17 | | rs971649 | | 8 | | 124732275 | | cg13177458 | | 124732088 | | 187 | |
| 33 | | 0.17 | | rs1139405 | | 17 | | 79478019 | | cg12793711 | | 79477853 | | 166 | |
|  | |  | |  | |  | |  | | cg26857837 | | 79477974 | | 45 | |
|  | |  | |  | |  | |  | | cg06713261 | | 79478173 | | 154 | |
|  | |  | |  | |  | |  | | cg15906692 | | 79478715 | | 696 | |
|  | |  | |  | |  | |  | | cg17317962 | | 79478839 | | 820 | |
| 37 | | 0.17 | | rs4687210 | | 3 | | 191046770 | | cg25342125 | | 191046380 | | 390 | |
|  | |  | |  | |  | |  | | cg19066520 | | 191046547 | | 223 | |
|  | |  | |  | |  | |  | | cg23528751 | | 191046721 | | 49 | |
|  | |  | |  | |  | |  | | cg14972328 | | 191046866 | | 96 | |
|  | |  | |  | |  | |  | | cg00473167 | | 191046872 | | 102 | |
|  | |  | |  | |  | |  | | cg17596905 | | 191046949 | | 179 | |
|  | |  | |  | |  | |  | | cg23817170 | | 191046997 | | 227 | |
|  | |  | |  | |  | |  | | cg26325444 | | 191047058 | | 288 | |
|  | |  | |  | |  | |  | | cg09688588 | | 191047460 | | 690 | |
| 38 | | 0.17 | | rs1774846 | | 1 | | 202994374 | | cg03639170 | | 202993923 | | 451 | |
|  | |  | |  | |  | |  | | cg00501542 | | 202995246 | | 872 | |
| 39 | | 0.17 | | rs12629627 | | 3 | | 73629548 | | cg14228812 | | 73630106 | | 558 | |
| 41 | | 0.17 | | rs6466131 | | 7 | | 106408569 | | cg09735627 | | 106407970 | | 599 | |
| 42 | | 0.17 | | rs1054497 | | 4 | | 174089048 | | cg27433088 | | 174089019 | | 29 | |
|  | |  | |  | |  | |  | | cg10817093 | | 174089402 | | 354 | |
|  | |  | |  | |  | |  | | cg06965122 | | 174089515 | | 467 | |
|  | |  | |  | |  | |  | | cg05897918 | | 174089748 | | 700 | |
|  | |  | |  | |  | |  | | cg18029791 | | 174089755 | | 707 | |
|  | |  | |  | |  | |  | | cg08422745 | | 174089978 | | 930 | |
| 43 | | 0.17 | | rs17366322 | | 2 | | 11272784 | | cg15704155 | | 11272399 | | 385 | |
|  | |  | |  | |  | |  | | cg09045196 | | 11272424 | | 360 | |
|  | |  | |  | |  | |  | | cg10334385 | | 11272868 | | 84 | |
|  | |  | |  | |  | |  | | cg27641709 | | 11272899 | | 115 | |
|  | |  | |  | |  | |  | | cg05517905 | | 11273075 | | 291 | |
|  | |  | |  | |  | |  | | cg12453288 | | 11273229 | | 445 | |
|  | |  | |  | |  | |  | | cg03251967 | | 11273291 | | 507 | |
| 44 | | 0.17 | | rs7305595 | | 12 | | 3562234 | | cg23794848 | | 3563020 | | 786 | |
| 45 | | 0.16 | | rs3762199 | | 20 | | 18487792 | | cg26234223 | | 18487771 | | 21 | |
|  | |  | |  | |  | |  | | cg01866064 | | 18487951 | | 159 | |
|  | |  | |  | |  | |  | | cg27634549 | | 18487976 | | 184 | |
|  | |  | |  | |  | |  | | cg23490046 | | 18487999 | | 207 | |
|  | |  | |  | |  | |  | | cg15613982 | | 18488017 | | 225 | |
|  | |  | |  | |  | |  | | cg16156954 | | 18488094 | | 302 | |
|  | |  | |  | |  | |  | | cg07973146 | | 18488185 | | 393 | |
|  | |  | |  | |  | |  | | cg02081051 | | 18488312 | | 520 | |
|  | |  | |  | |  | |  | | cg22243844 | | 18488366 | | 574 | |
|  | |  | |  | |  | |  | | cg13313269 | | 18488622 | | 830 | |
| 51 | | 0.16 | | rs10883857 | | 10 | | 105127587 | | cg14125903 | | 105126693 | | 894 | |
|  | |  | |  | |  | |  | | cg10301212 | | 105126702 | | 885 | |
|  | |  | |  | |  | |  | | cg20188621 | | 105127192 | | 395 | |
|  | |  | |  | |  | |  | | cg11667101 | | 105127581 | | 6 | |
|  | |  | |  | |  | |  | | cg18586343 | | 105127615 | | 28 | |
|  | |  | |  | |  | |  | | cg20684973 | | 105127632 | | 45 | |
|  | |  | |  | |  | |  | | cg18732587 | | 105127670 | | 83 | |
|  | |  | |  | |  | |  | | cg25713684 | | 105127687 | | 100 | |
|  | |  | |  | |  | |  | | cg16500454 | | 105127701 | | 114 | |
|  | |  | |  | |  | |  | | cg01360325 | | 105127811 | | 224 | |
|  | |  | |  | |  | |  | | cg26978691 | | 105128013 | | 426 | |
|  | |  | |  | |  | |  | | cg00245789 | | 105128183 | | 596 | |
|  | |  | |  | |  | |  | | cg23418095 | | 105128462 | | 875 | |
| 52 | | 0.16 | | rs1237543 | | 13 | | 24739364 | | cg03903831 | | 24739249 | | 115 | |
| 57 | | 0.16 | | rs1673087 | | 19 | | 37264190 | | cg05616754 | | 37263860 | | 330 | |
|  | |  | |  | |  | |  | | cg03430717 | | 37263925 | | 265 | |
|  | |  | |  | |  | |  | | cg27080211 | | 37263997 | | 193 | |
| 59 | | 0.16 | | rs6713344 | | 2 | | 112814045 | | cg09071112 | | 112813174 | | 871 | |
|  | |  | |  | |  | |  | | cg06723287 | | 112813613 | | 432 | |
|  | |  | |  | |  | |  | | cg11931949 | | 112814041 | | 4 | |
|  | |  | |  | |  | |  | | cg19917083 | | 112814452 | | 407 | |
| 62 | | 0.16 | | rs266388 | | 15 | | 67239366 | | cg09482748 | | 67239225 | | 141 | |
| 64 | | 0.16 | | rs9331942 | | 8 | | 27455114 | | cg01572979 | | 27455004 | | 110 | |
| 65 | | 0.15 | | rs2735971 | | 11 | | 2021649 | | cg27372170 | | 2021103 | | 546 | |
|  | |  | |  | |  | |  | | cg16574793 | | 2022324 | | 675 | |
|  | |  | |  | |  | |  | | cg09452478 | | 2022386 | | 737 | |
| 72 | | 0.15 | | rs11089197 | | 22 | | 18121334 | | cg16048163 | | 18121127 | | 207 | |
|  | |  | |  | |  | |  | | cg03887534 | | 18121194 | | 140 | |
|  | |  | |  | |  | |  | | cg24160660 | | 18121281 | | 53 | |
|  | |  | |  | |  | |  | | cg19898043 | | 18121309 | | 25 | |
|  | |  | |  | |  | |  | | cg23077364 | | 18121349 | | 15 | |
|  | |  | |  | |  | |  | | cg10625092 | | 18121415 | | 81 | |
|  | |  | |  | |  | |  | | cg24756391 | | 18121424 | | 90 | |
|  | |  | |  | |  | |  | | cg16593229 | | 18122303 | | 969 | |
| 73 | | 0.15 | | rs12085639 | | 1 | | 94478293 | | cg26904308 | | 94477653 | | 640 | |
| 75 | | 0.15 | | rs553046 | | 7 | | 140776401 | | cg11344121 | | 140776653 | | 252 | |
| 79 | | 0.15 | | rs986912 | | 3 | | 124284055 | | cg06218523 | | 124284219 | | 164 | |
| 82 | | 0.15 | | rs7166128 | | 15 | | 66946530 | | cg24382249 | | 66947171 | | 641 | |
|  | |  | |  | |  | |  | | cg09827761 | | 66947392 | | 862 | |
| 85 | | 0.15 | | rs17146982 | | 7 | | 22528568 | | cg10016647 | | 22528033 | | 535 | |
| 88 | | 0.15 | | rs10147026 | | 14 | | 62523148 | | cg25133706 | | 62522891 | | 257 | |
|  | |  | |  | |  | |  | | cg18261657 | | 62523785 | | 637 | |
| 89 | | 0.15 | | rs2244352 | | 21 | | 40757973 | | cg01322405 | | 40757317 | | 656 | |
|  | |  | |  | |  | |  | | cg00606841 | | 40757691 | | 282 | |
|  | |  | |  | |  | |  | | cg22858667 | | 40757750 | | 223 | |
|  | |  | |  | |  | |  | | cg26710963 | | 40757899 | | 74 | |
|  | |  | |  | |  | |  | | cg09916765 | | 40758208 | | 235 | |
|  | |  | |  | |  | |  | | cg21297395 | | 40758325 | | 352 | |
|  | |  | |  | |  | |  | | cg21459460 | | 40758346 | | 373 | |
| 94 | | 0.15 | | rs10499772 | | 7 | | 63766980 | | cg00834400 | | 63767240 | | 260 | |
|  | |  | |  | |  | |  | | cg09382800 | | 63767753 | | 773 | |
|  | |  | |  | |  | |  | | cg23849078 | | 63767841 | | 861 | |
| 96 | | 0.15 | | rs1870172 | | 10 | | 97990101 | | cg03281335 | | 97989507 | | 594 | |
| 97 | | 0.15 | | rs220030 | | 15 | | 25199768 | | cg21746532 | | 25198793 | | 975 | |
|  | |  | |  | |  | |  | | cg01432432 | | 25199028 | | 740 | |
|  | |  | |  | |  | |  | | cg08372135 | | 25199057 | | 711 | |
|  | |  | |  | |  | |  | | cg03858387 | | 25199164 | | 604 | |
|  | |  | |  | |  | |  | | cg02152271 | | 25199270 | | 498 | |
|  | |  | |  | |  | |  | | cg12298755 | | 25199713 | | 55 | |
|  | |  | |  | |  | |  | | cg18506672 | | 25200253 | | 485 | |
|  | |  | |  | |  | |  | | cg02125271 | | 25200406 | | 638 | |
|  | |  | |  | |  | |  | | cg26875073 | | 25200490 | | 722 | |
| 99 | | 0.14 | | rs10245302 | | 7 | | 70294323 | | cg18138580 | | 70294264 | | 59 | |
|  | |  | |  | |  | |  | | cg19560710 | | 70294511 | | 188 | |
|  | |  | |  | |  | |  | | cg25795398 | | 70294525 | | 202 | |
| 100 | | 0.14 | | rs2143346 | | 6 | | 26198449 | | cg23407396 | | 26197481 | | 968 | |
|  | |  | |  | |  | |  | | cg23601095 | | 26197514 | | 935 | |
|  | |  | |  | |  | |  | | cg16219491 | | 26197733 | | 716 | |
|  | |  | |  | |  | |  | | cg07329467 | | 26199075 | | 626 | |

| **Supplementary Table 12. Illumina 450K probes within 1kb of the top 100 cross-tissue ASM sites, as defined by average ASM score across individuals.** | | | | | | | |
| --- | --- | --- | --- | --- | --- | --- | --- |
| **Rank** | **ASM** | **SNP** | **Chr** | **Mapinfo SNP** | **CpG site** | **Mapinfo CpG** | **Distance** |
| 1 | 0.24 | rs10234308 | 7 | 22528590 | cg10016647 | 22528033 | 557 |
| 2 | 0.22 | rs12493005 | 3 | 176912899 | cg03356026 | 176912588 | 311 |
| 3 | 0.21 | rs1542180 | 2 | 177028767 | cg13316854 | 177027856 | 911 |
|  |  |  |  |  | cg06145336 | 177027931 | 836 |
|  |  |  |  |  | cg25938806 | 177028162 | 605 |
|  |  |  |  |  | cg12634591 | 177028606 | 161 |
|  |  |  |  |  | cg01128482 | 177028621 | 146 |
|  |  |  |  |  | cg09387749 | 177028680 | 87 |
|  |  |  |  |  | cg23981871 | 177028714 | 53 |
|  |  |  |  |  | cg10304824 | 177028804 | 37 |
|  |  |  |  |  | cg00005847 | 177029073 | 306 |
|  |  |  |  |  | cg24541426 | 177029459 | 692 |
|  |  |  |  |  | cg05021643 | 177029608 | 841 |
| 8 | 0.18 | rs4687210 | 3 | 191046770 | cg25342125 | 191046380 | 390 |
|  |  |  |  |  | cg19066520 | 191046547 | 223 |
|  |  |  |  |  | cg23528751 | 191046721 | 49 |
|  |  |  |  |  | cg14972328 | 191046866 | 96 |
|  |  |  |  |  | cg00473167 | 191046872 | 102 |
|  |  |  |  |  | cg17596905 | 191046949 | 179 |
|  |  |  |  |  | cg23817170 | 191046997 | 227 |
|  |  |  |  |  | cg26325444 | 191047058 | 288 |
|  |  |  |  |  | cg09688588 | 191047460 | 690 |
| 11 | 0.17 | rs1003533 | 5 | 131755651 | cg03347934 | 131754928 | 723 |
| 14 | 0.17 | rs220030 | 15 | 25199768 | cg21746532 | 25198793 | 975 |
|  |  |  |  |  | cg01432432 | 25199028 | 740 |
|  |  |  |  |  | cg08372135 | 25199057 | 711 |
|  |  |  |  |  | cg03858387 | 25199164 | 604 |
|  |  |  |  |  | cg02152271 | 25199270 | 498 |
|  |  |  |  |  | cg12298755 | 25199713 | 55 |
|  |  |  |  |  | cg18506672 | 25200253 | 485 |
|  |  |  |  |  | cg02125271 | 25200406 | 638 |
|  |  |  |  |  | cg26875073 | 25200490 | 722 |
| 19 | 0.17 | rs3121125 | 1 | 145382124 | cg23685870 | 145382340 | 216 |
|  |  |  |  |  | cg07117663 | 145382963 | 839 |
| 21 | 0.16 | rs17366322 | 2 | 11272784 | cg15704155 | 11272399 | 385 |
|  |  |  |  |  | cg09045196 | 11272424 | 360 |
|  |  |  |  |  | cg10334385 | 11272868 | 84 |
|  |  |  |  |  | cg27641709 | 11272899 | 115 |
|  |  |  |  |  | cg05517905 | 11273075 | 291 |
|  |  |  |  |  | cg12453288 | 11273229 | 445 |
|  |  |  |  |  | cg03251967 | 11273291 | 507 |
| 25 | 0.16 | rs3762199 | 20 | 18487792 | cg26234223 | 18487771 | 21 |
|  |  |  |  |  | cg01866064 | 18487951 | 159 |
|  |  |  |  |  | cg27634549 | 18487976 | 184 |
|  |  |  |  |  | cg23490046 | 18487999 | 207 |
|  |  |  |  |  | cg15613982 | 18488017 | 225 |
|  |  |  |  |  | cg16156954 | 18488094 | 302 |
|  |  |  |  |  | cg07973146 | 18488185 | 393 |
|  |  |  |  |  | cg02081051 | 18488312 | 520 |
|  |  |  |  |  | cg22243844 | 18488366 | 574 |
|  |  |  |  |  | cg13313269 | 18488622 | 830 |
| 26 | 0.16 | rs6713344 | 2 | 112814045 | cg09071112 | 112813174 | 871 |
|  |  |  |  |  | cg06723287 | 112813613 | 432 |
|  |  |  |  |  | cg11931949 | 112814041 | 4 |
|  |  |  |  |  | cg19917083 | 112814452 | 407 |
| 29 | 0.16 | rs3098382 | 5 | 71462632 | cg20954533 | 71462729 | 97 |
| 30 | 0.16 | rs17312325 | X | 68442113 | cg00427681 | 68441389 | 724 |
|  |  |  |  |  | cg22337892 | 68441690 | 423 |
|  |  |  |  |  | cg03350825 | 68442327 | 214 |
| 37 | 0.15 | rs1237543 | 13 | 24739364 | cg03903831 | 24739249 | 115 |
| 38 | 0.15 | rs7247675 | 19 | 28285519 | cg17155612 | 28284698 | 821 |
|  |  |  |  |  | cg02942594 | 28284741 | 778 |
|  |  |  |  |  | cg03170611 | 28284813 | 706 |
|  |  |  |  |  | cg22473312 | 28284823 | 696 |
|  |  |  |  |  | cg08220598 | 28284952 | 567 |
|  |  |  |  |  | cg17871739 | 28284973 | 546 |
|  |  |  |  |  | cg24415565 | 28284988 | 531 |
|  |  |  |  |  | cg25246092 | 28285128 | 391 |
|  |  |  |  |  | cg03751272 | 28285308 | 211 |
|  |  |  |  |  | cg06949053 | 28285395 | 124 |
| 39 | 0.15 | rs986912 | 3 | 124284055 | cg06218523 | 124284219 | 164 |
| 40 | 0.15 | rs7210715 | 17 | 7196390 | cg00901493 | 7197375 | 985 |
| 42 | 0.15 | rs7305595 | 12 | 3562234 | cg23794848 | 3563020 | 786 |
| 46 | 0.15 | rs2244352 | 21 | 40757973 | cg01322405 | 40757317 | 656 |
|  |  |  |  |  | cg00606841 | 40757691 | 282 |
|  |  |  |  |  | cg22858667 | 40757750 | 223 |
|  |  |  |  |  | cg26710963 | 40757899 | 74 |
|  |  |  |  |  | cg09916765 | 40758208 | 235 |
|  |  |  |  |  | cg21297395 | 40758325 | 352 |
|  |  |  |  |  | cg21459460 | 40758346 | 373 |
| 47 | 0.15 | rs7073504 | 10 | 60271987 | cg07567376 | 60271505 | 482 |
|  |  |  |  |  | cg09696939 | 60272079 | 92 |
|  |  |  |  |  | cg07857251 | 60272754 | 767 |
|  |  |  |  |  | cg19940537 | 60272938 | 951 |
| 49 | 0.15 | rs10883857 | 10 | 105127587 | cg14125903 | 105126693 | 894 |
|  |  |  |  |  | cg10301212 | 105126702 | 885 |
|  |  |  |  |  | cg20188621 | 105127192 | 395 |
|  |  |  |  |  | cg11667101 | 105127581 | 6 |
|  |  |  |  |  | cg18586343 | 105127615 | 28 |
|  |  |  |  |  | cg20684973 | 105127632 | 45 |
|  |  |  |  |  | cg18732587 | 105127670 | 83 |
|  |  |  |  |  | cg25713684 | 105127687 | 100 |
|  |  |  |  |  | cg16500454 | 105127701 | 114 |
|  |  |  |  |  | cg01360325 | 105127811 | 224 |
|  |  |  |  |  | cg26978691 | 105128013 | 426 |
|  |  |  |  |  | cg00245789 | 105128183 | 596 |
|  |  |  |  |  | cg23418095 | 105128462 | 875 |
| 52 | 0.15 | rs17146982 | 7 | 22528568 | cg10016647 | 22528033 | 535 |
| 55 | 0.14 | rs1139405 | 17 | 79478019 | cg12793711 | 79477853 | 166 |
|  |  |  |  |  | cg26857837 | 79477974 | 45 |
|  |  |  |  |  | cg06713261 | 79478173 | 154 |
|  |  |  |  |  | cg15906692 | 79478715 | 696 |
|  |  |  |  |  | cg17317962 | 79478839 | 820 |
| 56 | 0.14 | rs971649 | 8 | 124732275 | cg13177458 | 124732088 | 187 |
| 60 | 0.14 | rs2143346 | 6 | 26198449 | cg23407396 | 26197481 | 968 |
|  |  |  |  |  | cg23601095 | 26197514 | 935 |
|  |  |  |  |  | cg16219491 | 26197733 | 716 |
|  |  |  |  |  | cg07329467 | 26199075 | 626 |
| 64 | 0.14 | rs9331942 | 8 | 27455114 | cg01572979 | 27455004 | 110 |
| 66 | 0.14 | rs6874600 | 5 | 73945738 | cg20658167 | 73944869 | 869 |
| 76 | 0.14 | rs1673087 | 19 | 37264190 | cg05616754 | 37263860 | 330 |
|  |  |  |  |  | cg03430717 | 37263925 | 265 |
|  |  |  |  |  | cg27080211 | 37263997 | 193 |
| 78 | 0.14 | rs6548026 | 2 | 30872366 | cg08120035 | 30872901 | 535 |
| 81 | 0.14 | rs10499772 | 7 | 63766980 | cg00834400 | 63767240 | 260 |
|  |  |  |  |  | cg09382800 | 63767753 | 773 |
|  |  |  |  |  | cg23849078 | 63767841 | 861 |
| 89 | 0.13 | rs7185244 | 16 | 86546887 | cg08142918 | 86546374 | 513 |
|  |  |  |  |  | cg03697918 | 86546628 | 259 |
|  |  |  |  |  | cg24908603 | 86546631 | 256 |
|  |  |  |  |  | cg07056644 | 86546785 | 102 |
|  |  |  |  |  | cg27453745 | 86546938 | 51 |
|  |  |  |  |  | cg09338251 | 86546979 | 92 |
|  |  |  |  |  | cg06834912 | 86547203 | 316 |
|  |  |  |  |  | cg04787888 | 86547322 | 435 |
|  |  |  |  |  | cg01243371 | 86547386 | 499 |
|  |  |  |  |  | cg00551679 | 86547530 | 643 |
|  |  |  |  |  | cg02783918 | 86547544 | 657 |
| 90 | 0.13 | rs10454027 | 13 | 100259376 | cg22853485 | 100258524 | 852 |
|  |  |  |  |  | cg12175106 | 100258589 | 787 |
|  |  |  |  |  | cg01726399 | 100258641 | 735 |
|  |  |  |  |  | cg10972431 | 100258753 | 623 |
|  |  |  |  |  | cg02168723 | 100258760 | 616 |
|  |  |  |  |  | cg27435467 | 100258954 | 422 |
|  |  |  |  |  | cg20863393 | 100259188 | 188 |
|  |  |  |  |  | cg24980005 | 100259219 | 157 |
|  |  |  |  |  | cg23862604 | 100259302 | 74 |
| 94 | 0.13 | rs10108150 | 8 | 145687475 | cg13071208 | 145686887 | 588 |
|  |  |  |  |  | cg17328964 | 145687451 | 24 |
|  |  |  |  |  | cg15189104 | 145687562 | 87 |
|  |  |  |  |  | cg02108130 | 145687727 | 252 |
|  |  |  |  |  | cg25390352 | 145688187 | 712 |
|  |  |  |  |  | cg22601688 | 145688206 | 731 |
| 98 | 0.13 | rs8179387 | 1 | 22290156 | cg27522078 | 22290005 | 151 |
| 99 | 0.13 | rs9645876 | 13 | 112330861 | cg06567836 | 112330930 | 69 |
|  |  |  |  |  | cg09984047 | 112330954 | 93 |
|  |  |  |  |  | cg15453778 | 112331089 | 228 |
|  |  |  |  |  | cg06996147 | 112331836 | 975 |

| **Supplementary Table 13. Illumina 450K probes within 1kb of the top 100 tissue-specific ASM SNPs as defined by the high variability of ASM scores across tissues.** | | | | | | | |
| --- | --- | --- | --- | --- | --- | --- | --- |
| **Rank** | **ASM SD** | **SNP** | **Chr** | **Mapinfo SNP** | **CpG site** | **Mapinfo CpG** | **Distance** |
| 2 | 0.15 | rs927000 | 20 | 43598705 | cg17104026 | 43598376 | 329 |
| 17 | 0.13 | rs7959070 | 12 | 92805682 | cg25400182 | 92806045 | 363 |
| 18 | 0.12 | rs8032903 | 15 | 25980875 | cg16651441 | 25980367 | 508 |
|  |  |  |  |  | cg02747151 | 25981177 | 302 |
|  |  |  |  |  | cg09978546 | 25981255 | 380 |
|  |  |  |  |  | cg14216870 | 25981322 | 447 |
| 23 | 0.12 | rs6853895 | 4 | 38070899 | cg24826020 | 38070998 | 99 |
| 24 | 0.12 | rs7158663 | 14 | 101319424 | cg19509303 | 101318703 | 721 |
|  |  |  |  |  | cg07460524 | 101319526 | 102 |
| 25 | 0.12 | rs10783269 | 12 | 48995983 | cg18467358 | 48995259 | 724 |
| 27 | 0.12 | rs12510921 | 4 | 185189278 | cg04960065 | 185189300 | 22 |
| 29 | 0.11 | rs10481354 | 8 | 1704030 | cg19131313 | 1704013 | 17 |
|  |  |  |  |  | cg17159058 | 1704075 | 45 |
| 31 | 0.11 | rs1003533 | 5 | 131755651 | cg03347934 | 131754928 | 723 |
| 33 | 0.11 | rs2252397 | 7 | 140225750 | cg01499591 | 140226367 | 617 |
| 38 | 0.11 | rs12978286 | 19 | 17861868 | cg26307105 | 17861017 | 851 |
|  |  |  |  |  | cg02639359 | 17862017 | 149 |
|  |  |  |  |  | cg08206267 | 17862104 | 236 |
|  |  |  |  |  | cg24783510 | 17862150 | 282 |
|  |  |  |  |  | cg11737334 | 17862210 | 342 |
|  |  |  |  |  | cg22317846 | 17862385 | 517 |
| 46 | 0.11 | rs10411704 | 19 | 35800662 | cg02776658 | 35800589 | 73 |
|  |  |  |  |  | cg22238209 | 35800743 | 81 |
|  |  |  |  |  | cg07918620 | 35800925 | 263 |
|  |  |  |  |  | cg15761414 | 35801014 | 352 |
| 49 | 0.11 | rs1009014 | 6 | 158487143 | cg18559896 | 158487541 | 398 |
|  |  |  |  |  | cg03544657 | 158487564 | 421 |
|  |  |  |  |  | cg07549551 | 158487608 | 465 |
| 51 | 0.10 | rs3098382 | 5 | 71462632 | cg20954533 | 71462729 | 97 |
| 56 | 0.10 | rs9469529 | 6 | 33587443 | cg08355863 | 33587496 | 53 |
|  |  |  |  |  | cg24603235 | 33588219 | 776 |
|  |  |  |  |  | cg19889152 | 33588278 | 835 |
|  |  |  |  |  | cg14639225 | 33588302 | 859 |
| 62 | 0.10 | rs14067 | 13 | 114110660 | cg05314639 | 114109729 | 931 |
|  |  |  |  |  | cg04870949 | 114109857 | 803 |
|  |  |  |  |  | cg03557916 | 114109949 | 711 |
| 64 | 0.10 | rs11603592 | 11 | 20023522 | cg09261702 | 20023953 | 431 |
| 70 | 0.10 | rs7268068 | 20 | 62199258 | cg23352030 | 62198469 | 789 |
|  |  |  |  |  | cg15262954 | 62198872 | 386 |
|  |  |  |  |  | cg17593958 | 62199034 | 224 |
|  |  |  |  |  | cg11779113 | 62199156 | 102 |
|  |  |  |  |  | cg06064964 | 62199181 | 77 |
|  |  |  |  |  | cg09844573 | 62199190 | 68 |
|  |  |  |  |  | cg17864091 | 62200091 | 833 |
|  |  |  |  |  | cg04300115 | 62200199 | 941 |
| 83 | 0.10 | rs17539168 | 9 | 134485090 | cg14485643 | 134484581 | 509 |
| 84 | 0.10 | rs16843471 | 1 | 198590421 | cg10768932 | 198590738 | 317 |
| 89 | 0.10 | rs1774846 | 1 | 202994374 | cg03639170 | 202993923 | 451 |
|  |  |  |  |  | cg00501542 | 202995246 | 872 |
| 95 | 0.10 | rs10861495 | 12 | 106142981 | cg23539745 | 106142108 | 873 |

| **Supplementary Table 14. Illumina 450K probes within 1kb of the top 100 variable ASM sites, as defined by average range of ASM scores between individuals across tissues.** | | | | | | | |
| --- | --- | --- | --- | --- | --- | --- | --- |
| **Rank** | **ASM Range** | **SNP** | **Chr** | **Mapinfo SNP** | **CpG site** | **Mapinfo CG** | **Distance** |
| 2 | 0.30 | rs2244352 | 21 | 40757973 | cg01322405 | 40757317 | 656 |
|  |  |  |  |  | cg00606841 | 40757691 | 282 |
|  |  |  |  |  | cg22858667 | 40757750 | 223 |
|  |  |  |  |  | cg26710963 | 40757899 | 74 |
|  |  |  |  |  | cg09916765 | 40758208 | 235 |
|  |  |  |  |  | cg21297395 | 40758325 | 352 |
|  |  |  |  |  | cg21459460 | 40758346 | 373 |
| 3 | 0.25 | rs2346019 | 5 | 135415726 | cg11852404 | 135414858 | 868 |
|  |  |  |  |  | cg00308130 | 135415190 | 536 |
|  |  |  |  |  | cg15837280 | 135415258 | 468 |
|  |  |  |  |  | cg07158503 | 135415693 | 33 |
|  |  |  |  |  | cg04515200 | 135415762 | 36 |
|  |  |  |  |  | cg13581155 | 135415781 | 55 |
|  |  |  |  |  | cg11978884 | 135415819 | 93 |
|  |  |  |  |  | cg11608150 | 135415948 | 222 |
|  |  |  |  |  | cg06478886 | 135416029 | 303 |
|  |  |  |  |  | cg04481923 | 135416205 | 479 |
|  |  |  |  |  | cg18678645 | 135416331 | 605 |
|  |  |  |  |  | cg06536614 | 135416381 | 655 |
|  |  |  |  |  | cg26328633 | 135416394 | 668 |
|  |  |  |  |  | cg25340688 | 135416398 | 672 |
|  |  |  |  |  | cg26896946 | 135416405 | 679 |
|  |  |  |  |  | cg00124993 | 135416412 | 686 |
|  |  |  |  |  | cg08745965 | 135416529 | 803 |
|  |  |  |  |  | cg16615357 | 135416594 | 868 |
|  |  |  |  |  | cg18797653 | 135416613 | 887 |
| 7 | 0.19 | rs1209228 | 14 | 72231880 | cg15926477 | 72231668 | 212 |
| 13 | 0.18 | rs1695824 | 1 | 1365570 | cg04865726 | 1365911 | 341 |
|  |  |  |  |  | cg12407057 | 1366206 | 636 |
|  |  |  |  |  | cg11156891 | 1366274 | 704 |
| 18 | 0.17 | rs578597 | 9 | 104185258 | cg14048797 | 104186172 | 914 |
| 23 | 0.16 | rs3759917 | 15 | 92935447 | cg18646851 | 92934809 | 638 |
|  |  |  |  |  | cg08152839 | 92936232 | 785 |
|  |  |  |  |  | cg05501584 | 92936306 | 859 |
| 26 | 0.16 | rs11231646 | 11 | 63702635 | cg05766107 | 63702235 | 400 |
| 28 | 0.16 | rs1984713 | 4 | 47956556 | cg06587443 | 47955755 | 801 |
|  |  |  |  |  | cg19000186 | 47956011 | 545 |
| 33 | 0.16 | rs26835 | 16 | 2237549 | cg07094340 | 2236773 | 776 |
|  |  |  |  |  | cg10118998 | 2236938 | 611 |
|  |  |  |  |  | cg07109743 | 2237020 | 529 |
|  |  |  |  |  | cg08938612 | 2237221 | 328 |
| 35 | 0.16 | rs10952289 | 7 | 150524681 | cg05405275 | 150525013 | 332 |
| 50 | 0.15 | rs11611523 | 12 | 118018891 | cg05738715 | 118018768 | 123 |
| 51 | 0.15 | rs2400135 | 6 | 106253120 | cg01182171 | 106252695 | 425 |
| 54 | 0.15 | rs2377983 | 10 | 50352720 | cg14620932 | 50353359 | 639 |
| 69 | 0.14 | rs6779820 | 3 | 191046793 | cg25342125 | 191046380 | 413 |
|  |  |  |  |  | cg19066520 | 191046547 | 246 |
|  |  |  |  |  | cg23528751 | 191046721 | 72 |
|  |  |  |  |  | cg14972328 | 191046866 | 73 |
|  |  |  |  |  | cg00473167 | 191046872 | 79 |
|  |  |  |  |  | cg17596905 | 191046949 | 156 |
|  |  |  |  |  | cg23817170 | 191046997 | 204 |
|  |  |  |  |  | cg26325444 | 191047058 | 265 |
|  |  |  |  |  | cg09688588 | 191047460 | 667 |
| 71 | 0.14 | rs509062 | 20 | 56882238 | cg21532801 | 56881949 | 289 |
| 76 | 0.14 | rs8179356 | 1 | 100215981 | cg17222434 | 100215737 | 244 |
| 80 | 0.14 | rs7911580 | 10 | 7516306 | cg03421195 | 7517215 | 909 |
| 84 | 0.14 | rs2294490 | 1 | 15723843 | cg00582948 | 15724194 | 351 |
| 87 | 0.14 | rs4289794 | 8 | 141106204 | cg12844366 | 141105335 | 869 |
|  |  |  |  |  | cg00085199 | 141106596 | 392 |
|  |  |  |  |  | cg05524387 | 141107113 | 909 |
| 91 | 0.14 | rs6045166 | 20 | 18035837 | cg02113429 | 18036013 | 176 |
| 94 | 0.13 | rs12525580 | 6 | 74288782 | cg05488681 | 74289464 | 682 |
| 98 | 0.13 | rs3109398 | 3 | 182402101 | cg09391284 | 182401187 | 914 |
|  |  |  |  |  | cg20830867 | 182402080 | 21 |

| **Supplementary Table 15. Illumina 450K probes within 1kb of the top 100 variable ASM SNPs in whole blood as defined by range of ASM score between individuals.** | | | | | | | |
| --- | --- | --- | --- | --- | --- | --- | --- |
| **Rank** | **Blood range** | **SNP** | **Chr** | **Mapinfo SNP** | **CpG site** | **Mapinfo CpG** | **Distance** |
| 3 | 0.25 | rs509062 | 20 | 56882238 | cg21532801 | 56881949 | 289 |
| 4 | 0.24 | rs2244352 | 21 | 40757973 | cg01322405 | 40757317 | 656 |
|  |  |  |  |  | cg00606841 | 40757691 | 282 |
|  |  |  |  |  | cg22858667 | 40757750 | 223 |
|  |  |  |  |  | cg26710963 | 40757899 | 74 |
|  |  |  |  |  | cg09916765 | 40758208 | 235 |
|  |  |  |  |  | cg21297395 | 40758325 | 352 |
|  |  |  |  |  | cg21459460 | 40758346 | 373 |
| 6 | 0.23 | rs8179356 | 1 | 100215981 | cg17222434 | 100215737 | 244 |
| 8 | 0.23 | rs2346019 | 5 | 135415726 | cg11852404 | 135414858 | 868 |
|  |  |  |  |  | cg00308130 | 135415190 | 536 |
|  |  |  |  |  | cg15837280 | 135415258 | 468 |
|  |  |  |  |  | cg07158503 | 135415693 | 33 |
|  |  |  |  |  | cg04515200 | 135415762 | 36 |
|  |  |  |  |  | cg13581155 | 135415781 | 55 |
|  |  |  |  |  | cg11978884 | 135415819 | 93 |
|  |  |  |  |  | cg11608150 | 135415948 | 222 |
|  |  |  |  |  | cg06478886 | 135416029 | 303 |
|  |  |  |  |  | cg04481923 | 135416205 | 479 |
|  |  |  |  |  | cg18678645 | 135416331 | 605 |
|  |  |  |  |  | cg06536614 | 135416381 | 655 |
|  |  |  |  |  | cg26328633 | 135416394 | 668 |
|  |  |  |  |  | cg25340688 | 135416398 | 672 |
|  |  |  |  |  | cg26896946 | 135416405 | 679 |
|  |  |  |  |  | cg00124993 | 135416412 | 686 |
|  |  |  |  |  | cg08745965 | 135416529 | 803 |
|  |  |  |  |  | cg16615357 | 135416594 | 868 |
|  |  |  |  |  | cg18797653 | 135416613 | 887 |
| 10 | 0.22 | rs7694398 | 4 | 6733947 | cg00966522 | 6734722 | 775 |
| 17 | 0.20 | rs1890070 | 1 | 222628509 | cg15078329 | 222628324 | 185 |
|  |  |  |  |  | cg11661340 | 222628632 | 123 |
|  |  |  |  |  | cg17836114 | 222628702 | 193 |
| 19 | 0.20 | rs1984713 | 4 | 47956556 | cg06587443 | 47955755 | 801 |
|  |  |  |  |  | cg19000186 | 47956011 | 545 |
| 23 | 0.19 | rs10186949 | 2 | 237755950 | cg04787916 | 237755762 | 188 |
| 30 | 0.19 | rs849322 | 7 | 28331586 | cg26353161 | 28331380 | 206 |
| 38 | 0.18 | rs578597 | 9 | 104185258 | cg14048797 | 104186172 | 914 |
| 46 | 0.18 | rs165342 | 5 | 150884793 | cg22672639 | 150884813 | 20 |
| 62 | 0.17 | rs11231646 | 11 | 63702635 | cg05766107 | 63702235 | 400 |
| 64 | 0.17 | rs2072019 | 6 | 112460541 | cg12188410 | 112460467 | 74 |
| 66 | 0.17 | rs2357928 | 10 | 18549641 | cg25091573 | 18549036 | 605 |
|  |  |  |  |  | cg24422029 | 18549378 | 263 |
|  |  |  |  |  | cg14716734 | 18549397 | 244 |
|  |  |  |  |  | cg05205813 | 18549413 | 228 |
|  |  |  |  |  | cg09987620 | 18549439 | 202 |
|  |  |  |  |  | cg26803268 | 18549536 | 105 |
|  |  |  |  |  | cg18959207 | 18549686 | 45 |
|  |  |  |  |  | cg02635932 | 18549778 | 137 |
|  |  |  |  |  | cg14357089 | 18550033 | 392 |
|  |  |  |  |  | cg26576398 | 18550135 | 494 |
|  |  |  |  |  | cg15720089 | 18550223 | 582 |
| 86 | 0.17 | rs12938106 | 17 | 29926633 | cg00351813 | 29926184 | 449 |
|  |  |  |  |  | cg25208821 | 29926356 | 277 |
| 89 | 0.17 | rs7174808 | 15 | 52048251 | cg02253142 | 52048211 | 40 |
| 91 | 0.17 | rs1464172 | 3 | 124873295 | cg17478992 | 124873768 | 473 |
|  |  |  |  |  | cg01432055 | 124873848 | 553 |
| 92 | 0.17 | rs875981 | 11 | 130903093 | cg01464703 | 130903135 | 42 |
| 96 | 0.17 | rs10116248 | 9 | 132568500 | cg14222752 | 132569372 | 872 |

| **Supplementary Table 16. Illumina 450K probes within 1kb of the top 100 variable ASM SNPs in cerebellum as defined by range of ASM score between individuals.** | | | | | | | |
| --- | --- | --- | --- | --- | --- | --- | --- |
| **Rank** | **Cerebellum range** | **SNP** | **Chr** | **Mapinfo SNP** | **CpG site** | **Mapinfo CpG** | **Distance** |
| 1 | 0.36 | rs2244352 | 21 | 40757973 | cg01322405 | 40757317 | 656 |
|  |  |  |  |  | cg00606841 | 40757691 | 282 |
|  |  |  |  |  | cg22858667 | 40757750 | 223 |
|  |  |  |  |  | cg26710963 | 40757899 | 74 |
|  |  |  |  |  | cg09916765 | 40758208 | 235 |
|  |  |  |  |  | cg21297395 | 40758325 | 352 |
|  |  |  |  |  | cg21459460 | 40758346 | 373 |
| 8 | 0.25 | rs1695824 | 1 | 1365570 | cg04865726 | 1365911 | 341 |
|  |  |  |  |  | cg12407057 | 1366206 | 636 |
|  |  |  |  |  | cg11156891 | 1366274 | 704 |
| 11 | 0.24 | rs4289794 | 8 | 141106204 | cg12844366 | 141105335 | 869 |
|  |  |  |  |  | cg00085199 | 141106596 | 392 |
|  |  |  |  |  | cg05524387 | 141107113 | 909 |
| 13 | 0.24 | rs3759917 | 15 | 92935447 | cg18646851 | 92934809 | 638 |
|  |  |  |  |  | cg08152839 | 92936232 | 785 |
|  |  |  |  |  | cg05501584 | 92936306 | 859 |
| 15 | 0.23 | rs2346019 | 5 | 135415726 | cg11852404 | 135414858 | 868 |
|  |  |  |  |  | cg00308130 | 135415190 | 536 |
|  |  |  |  |  | cg15837280 | 135415258 | 468 |
|  |  |  |  |  | cg07158503 | 135415693 | 33 |
|  |  |  |  |  | cg04515200 | 135415762 | 36 |
|  |  |  |  |  | cg13581155 | 135415781 | 55 |
|  |  |  |  |  | cg11978884 | 135415819 | 93 |
|  |  |  |  |  | cg11608150 | 135415948 | 222 |
|  |  |  |  |  | cg06478886 | 135416029 | 303 |
|  |  |  |  |  | cg04481923 | 135416205 | 479 |
|  |  |  |  |  | cg18678645 | 135416331 | 605 |
|  |  |  |  |  | cg06536614 | 135416381 | 655 |
|  |  |  |  |  | cg26328633 | 135416394 | 668 |
|  |  |  |  |  | cg25340688 | 135416398 | 672 |
|  |  |  |  |  | cg26896946 | 135416405 | 679 |
|  |  |  |  |  | cg00124993 | 135416412 | 686 |
|  |  |  |  |  | cg08745965 | 135416529 | 803 |
|  |  |  |  |  | cg16615357 | 135416594 | 868 |
|  |  |  |  |  | cg18797653 | 135416613 | 887 |
| 18 | 0.23 | rs941483 | 14 | 94422623 | cg00839873 | 94421989 | 634 |
|  |  |  |  |  | cg18572214 | 94423192 | 569 |
|  |  |  |  |  | cg01956154 | 94423399 | 776 |
| 28 | 0.21 | rs578597 | 9 | 104185258 | cg14048797 | 104186172 | 914 |
| 35 | 0.20 | rs2377983 | 10 | 50352720 | cg14620932 | 50353359 | 639 |
| 40 | 0.19 | rs1209228 | 14 | 72231880 | cg15926477 | 72231668 | 212 |
| 42 | 0.19 | rs7426865 | 3 | 149058065 | cg06305422 | 149057820 | 245 |
| 60 | 0.18 | rs12412942 | 10 | 2357702 | cg23939236 | 2357224 | 478 |
|  |  |  |  |  | cg03163525 | 2357283 | 419 |
|  |  |  |  |  | cg17246535 | 2357363 | 339 |
|  |  |  |  |  | cg03946168 | 2357461 | 241 |
|  |  |  |  |  | cg18730194 | 2357587 | 115 |
| 68 | 0.18 | rs1708337 | 3 | 124886822 | cg24838136 | 124887734 | 912 |
| 76 | 0.17 | rs4253747 | 22 | 46613237 | cg06467025 | 46614169 | 932 |
| 77 | 0.17 | rs17146982 | 7 | 22528568 | cg10016647 | 22528033 | 535 |
| 88 | 0.17 | rs9348131 | 6 | 166850798 | cg11314174 | 166850928 | 130 |
|  |  |  |  |  | cg02624984 | 166851100 | 302 |
|  |  |  |  |  | cg21313980 | 166851372 | 574 |
| 90 | 0.17 | rs26835 | 16 | 2237549 | cg07094340 | 2236773 | 776 |
|  |  |  |  |  | cg10118998 | 2236938 | 611 |
|  |  |  |  |  | cg07109743 | 2237020 | 529 |
|  |  |  |  |  | cg08938612 | 2237221 | 328 |
| 96 | 0.17 | rs7911580 | 10 | 7516306 | cg03421195 | 7517215 | 909 |
| 97 | 0.17 | rs3735715 | 8 | 102679909 | cg25876227 | 102680096 | 187 |

| **Supplementary Table 17. Illumina 450K probes within 1kb of the top 100 variable ASM SNPs in cortex (BA9) as defined by range of ASM score between individuals.** | | | | | | | |
| --- | --- | --- | --- | --- | --- | --- | --- |
| **Rank** | **BA9 range** | **SNP** | **Chr** | **Mapinfo SNP** | **CpG site** | **Mapinfo CpG** | **Distance** |
| 3 | 0.30 | rs2346019 | 5 | 135415726 | cg11852404 | 135414858 | 868 |
|  |  |  |  |  | cg00308130 | 135415190 | 536 |
|  |  |  |  |  | cg15837280 | 135415258 | 468 |
|  |  |  |  |  | cg07158503 | 135415693 | 33 |
|  |  |  |  |  | cg04515200 | 135415762 | 36 |
|  |  |  |  |  | cg13581155 | 135415781 | 55 |
|  |  |  |  |  | cg11978884 | 135415819 | 93 |
|  |  |  |  |  | cg11608150 | 135415948 | 222 |
|  |  |  |  |  | cg06478886 | 135416029 | 303 |
|  |  |  |  |  | cg04481923 | 135416205 | 479 |
|  |  |  |  |  | cg18678645 | 135416331 | 605 |
|  |  |  |  |  | cg06536614 | 135416381 | 655 |
|  |  |  |  |  | cg26328633 | 135416394 | 668 |
|  |  |  |  |  | cg25340688 | 135416398 | 672 |
|  |  |  |  |  | cg26896946 | 135416405 | 679 |
|  |  |  |  |  | cg00124993 | 135416412 | 686 |
|  |  |  |  |  | cg08745965 | 135416529 | 803 |
|  |  |  |  |  | cg16615357 | 135416594 | 868 |
|  |  |  |  |  | cg18797653 | 135416613 | 887 |
| 4 | 0.29 | rs2244352 | 21 | 40757973 | cg01322405 | 40757317 | 656 |
|  |  |  |  |  | cg00606841 | 40757691 | 282 |
|  |  |  |  |  | cg22858667 | 40757750 | 223 |
|  |  |  |  |  | cg26710963 | 40757899 | 74 |
|  |  |  |  |  | cg09916765 | 40758208 | 235 |
|  |  |  |  |  | cg21297395 | 40758325 | 352 |
|  |  |  |  |  | cg21459460 | 40758346 | 373 |
| 9 | 0.24 | rs1209228 | 14 | 72231880 | cg15926477 | 72231668 | 212 |
| 10 | 0.23 | rs9357087 | 6 | 11216700 | cg05208582 | 11216594 | 106 |
| 11 | 0.22 | rs503755 | 2 | 135228060 | cg05670240 | 135227167 | 893 |
| 22 | 0.20 | rs17008180 | 4 | 106406083 | cg08987594 | 106405819 | 264 |
|  |  |  |  |  | cg20364776 | 106405850 | 233 |
| 33 | 0.19 | rs2377983 | 10 | 50352720 | cg14620932 | 50353359 | 639 |
| 35 | 0.19 | rs9395212 | 6 | 46726441 | cg10669279 | 46726850 | 409 |
| 47 | 0.18 | rs633644 | 21 | 44783063 | cg02260098 | 44782434 | 629 |
|  |  |  |  |  | cg25191041 | 44782470 | 593 |
|  |  |  |  |  | cg14081667 | 44782497 | 566 |
|  |  |  |  |  | cg19318330 | 44782872 | 191 |
| 49 | 0.18 | rs985462 | 10 | 127769598 | cg19997196 | 127769491 | 107 |
|  |  |  |  |  | cg08295661 | 127769903 | 305 |
| 51 | 0.18 | rs10952289 | 7 | 150524681 | cg05405275 | 150525013 | 332 |
| 54 | 0.18 | rs17573298 | 16 | 78218016 | cg04344190 | 78218445 | 429 |
| 56 | 0.18 | rs6577101 | 13 | 113293717 | cg14227911 | 113292824 | 893 |
|  |  |  |  |  | cg07014973 | 113292951 | 766 |
| 57 | 0.18 | rs12525580 | 6 | 74288782 | cg05488681 | 74289464 | 682 |
| 58 | 0.18 | rs4784910 | 16 | 58298471 | cg08638917 | 58299153 | 682 |
| 72 | 0.18 | rs17130554 | 1 | 68653137 | cg22850128 | 68654112 | 975 |
| 76 | 0.18 | rs17432433 | 15 | 37174748 | cg17921924 | 37174376 | 372 |
|  |  |  |  |  | cg22444584 | 37174661 | 87 |
|  |  |  |  |  | cg27042266 | 37175007 | 259 |
|  |  |  |  |  | cg16890051 | 37175029 | 281 |
|  |  |  |  |  | cg12288941 | 37175117 | 369 |
| 90 | 0.17 | rs10886631 | 10 | 85325650 | cg12554194 | 85324700 | 950 |
|  |  |  |  |  | cg04567661 | 85325001 | 649 |
|  |  |  |  |  | cg24127335 | 85325062 | 588 |
| 92 | 0.17 | rs11135514 | 5 | 96790262 | cg21913787 | 96789385 | 877 |
| 98 | 0.17 | rs2070682 | 7 | 100777267 | cg17968347 | 100777740 | 473 |

| **Supplementary Table 18. Sample information.** Shown is demographic information for individuals profiled for ASM using our MSNP protocol and for DNA methylation using the Illumina Infinium HumanMethylation450 BeadChip (450K). PMD = Post-mortem delay (h). | | | | | | | | | | | |
| --- | --- | --- | --- | --- | --- | --- | --- | --- | --- | --- | --- |
|  | **Demographics** | | | **Tissue** |  |  |  |  |  |  |  |
| **ID** | **Sex** | **Age at death** | **PMD** | **BA8** | **BA9** | **BA10** | **BA 17** | **BA21** | **BA28/34** | **Cerebellum** | **Blood** |
| 1 | F | 82 | 43 | MSNP | MSNP | MSNP |  | MSNP | MSNP | MSNP | MSNP |
|  |  |  |  |  | 450K |  |  | 450K | 450K | 450K | 450K |
| 2 | M | 78 | NA | MSNP | MSNP | MSNP |  |  | MSNP | MSNP | MSNP |
|  |  |  |  |  | 450K |  |  | 450K | 450K | 450K | 450K |
| 3 | F | 92 | 17 | MSNP | MSNP | MSNP | MSNP | MSNP | MSNP | MSNP | MSNP |
|  |  |  |  |  | 450K |  |  | 450K | 450K | 450K | 450K |
| 4 | F | 82 | 13 |  | 450K |  |  | 450K | 450K | 450K | 450K |
| 5 | F | 81 | 17 |  | 450K |  |  | 450K | 450K | 450K |  |
| 6 | M | 86 | 7 |  | 450K |  |  | 450K | 450K | 450K | 450K |
| 7 | F | 80 | 3 |  | 450K |  |  | 450K | 450K | 450K |  |
| 8 | F | 55 | 12 |  | 450K |  |  | 450K | 450K | 450K |  |
| 9 | M | 79 | 47 |  | 450K |  |  | 450K | 450K | 450K |  |
| 10 | M | 80 | 21 |  | 450K |  |  | 450K | 450K | 450K |  |
| 11 | M | 86 | 6 |  | 450K |  |  | 450K | 450K | 450K |  |
| 12 | F | 87 | 22 |  | 450K |  |  | 450K |  | 450K |  |
| 13 | F | 68 | 9 |  |  |  |  | 450K |  | 450K |  |
| 14 | M | 59 | 50 |  | 450K |  |  | 450K | 450K | 450K |  |
| 15 | F | 80 | 48 |  | 450K |  |  | 450K | 450K | 450K |  |
| 16 | M | 40 | 40 |  |  |  |  | 450K |  | 450K |  |
| 17 | F | 55 | 95 |  | 450K |  |  | 450K |  | 450K |  |
| 18 | F | 73 | 70 |  | 450K |  |  | 450K |  |  |  |
| 19 | M | 80 | 60 |  | 450K |  |  | 450K | 450K | 450K |  |
| 20 | F | 90 | 50 |  | 450K |  |  | 450K | 450K | 450K |  |
| 21 | M | 81 | 18 |  | 450K |  |  | 450K | 450K | 450K |  |
| 22 | M | 66 | 52 |  | 450K |  |  | 450K | 450K | 450K |  |
| 23 | F | 88 | 6 |  | 450K |  |  | 450K | 450K | 450K |  |
| 24 | M | 86 | 21 |  | 450K |  |  | 450K | 450K | 450K | 450K |
| 25 | F | 90 | 56 |  | 450K |  |  | 450K | 450K | 450K | 450K |
| 26 | F | 92 | 23 |  | 450K |  |  | 450K | 450K | 450K | 450K |
| 27 | M | 97 | 44 |  | 450K |  |  | 450K | 450K | 450K |  |
| 28 | M | 74 | 23 |  | 450K |  |  | 450K | 450K | 450K |  |
| 29 | M | 77 | 11 |  | 450K |  |  | 450K | 450K | 450K |  |
| 30 | M | 82 | 47 |  | 450K |  |  | 450K | 450K | 450K |  |
| 31 | M | 73 | 23 |  | 450K |  |  | 450K | 450K | 450K |  |
| 32 | M | 95 | 26 |  | 450K |  |  | 450K | 450K | 450K |  |
| 33 | F | 79 | 56 |  | 450K |  |  | 450K | 450K | 450K |  |
| 34 | F | 91 | 74 |  | 450K |  |  | 450K | 450K | 450K |  |
| 35 | M | NA | NA |  |  |  |  |  |  |  | 450K |
| 36 | F | NA | NA |  |  |  |  |  |  |  | 450K |
| 37 | F | NA | NA |  |  |  |  |  |  |  | 450K |
| 38 | F | NA | NA |  |  |  |  |  |  |  | 450K |
| 39 | M | NA | NA |  |  |  |  |  |  |  | 450K |

| **Supplementary Table 19. Number of informative loci interrogated by the MSNP protocol for each sample.** | | | | | | | | | |
| --- | --- | --- | --- | --- | --- | --- | --- | --- | --- |
|  | **BA8** | **BA9** | **BA10** | **BA17** | **BA21** | **BA28/34** | **Cerebellum** | **Blood** | **Total** |
| Individual 1 | 110004 | 110004 | 110004 |  | 110004 | 110004 | 110004 | 110004 | 110004 |
| Individual 2 | 107649 | 107649 | 107649 |  |  | 107649 | 107649 | 107649 | 107649 |
| Individual 3 | 108942 | 108942 | 108942 | 108942 | 108942 | 108942 | 108942 | 108942 | 108942 |
| Total | 220449 | 220449 | 220449 | 108942 | 220449 | 177162 | 220449 | 220449 |  |

| **Supplementary Table 20. Primers used for clonal bisulfite sequencing.** | | |
| --- | --- | --- |
| **Assay** | **Forward Primer** | **Reverse Primer** |
| SNP_A-4255628 (rs959246) | TGGTTTGGTAGGTGTTAGGTATTTAG | CTCCCTATTCCCCAATAAAAATTTA |
| SNP_A-2273834 (rs2252267) | TTTTTGAGTGATTGGTTATTAGAAA | CCCAAAAAAACTAAAATTCCCTAAC |
